# Differentiation of Dihydroxylated Vitamin D_3_ Isomers Using Tandem Mass Spectrometry

**DOI:** 10.1021/jasms.2c00085

**Published:** 2022-05-13

**Authors:** Anisha Haris, Yuko P. Y. Lam, Christopher A. Wootton, Alina Theisen, Bryan P. Marzullo, Pascal Schorr, Dietrich A. Volmer, Peter B. O’Connor

**Affiliations:** † Department of Chemistry, University of Warwick, Coventry CV4 7AL, U.K.; ‡ Institut für Chemie, Humboldt-Universität zu Berlin, 12489 Berlin, Germany

## Abstract

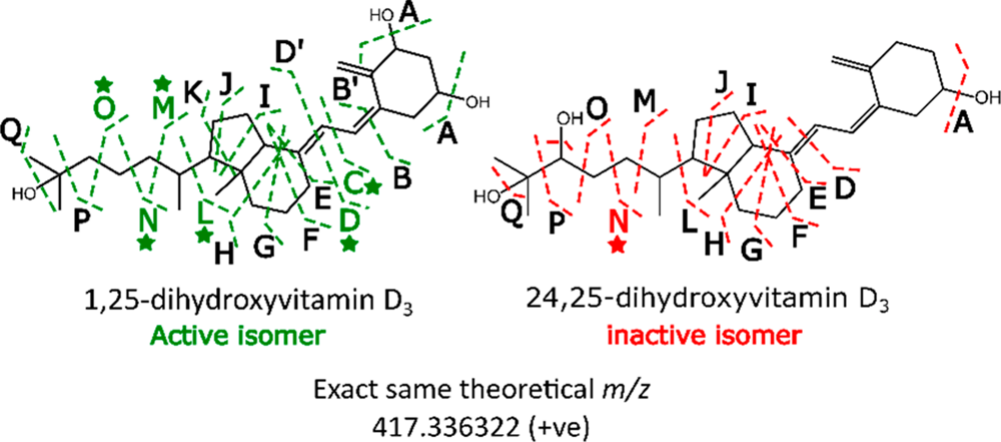

Vitamin D compounds are a group of secosteroids derived from cholesterol that are vital for maintaining bone health in humans. Recent studies have shown extraskeletal effects of vitamin D, involving vitamin D metabolites such as the dihydroxylated vitamin D_3_ compounds 1,25-dihydroxyvitamin D_3_ and 24,25-dihydroxyvitamin D_3_. Differentiation and characterization of these isomers by mass spectrometry can be challenging due to the zero-mass difference and minor structural differences between them. The isomers usually require separation by liquid chromatography (LC) prior to mass spectrometry, which adds extra complexity to the analysis. Herein, we investigated and revisited the use of fragmentation methods such as collisional induced dissociation (CID), infrared multiphoton dissociation (IRMPD), electron induced dissociation (EID), and ultraviolet photodissociation (UVPD), available on a 12T Fourier transform ion cyclotron resonance mass spectrometer (FT-ICR MS) to generate characteristic fragments for the dihydroxylated vitamin D_3_ isomers that can be used to distinguish between them. Isomer-specific fragments were observed for the 1,25-dihydroxyvitamin D_3_, which were clearly absent in the 24,25-dihydroxyvitamin D_3_ MS/MS spectra using all fragmentation methods mentioned above. The fragments generated due to cleavage of the C-6/C-7 bond in the 1,25-dihydroxyvitamin D_3_ compound demonstrate that the fragile OH groups were retained during fragmentation, thus enabling differentiation between the two dihydroxylated vitamin D_3_ isomers without the need for prior chromatographic separation or derivatization.

## Introduction

Vitamin D compounds comprise a class of fat-soluble secosteroids exhibiting some biological activity. Vitamin D_3_ (VD_3_) is primarily formed in the skin of mammals via photosynthesis, and it is widely known to regulate the amount of important minerals such as phosphate and calcium in the body.^1^ These nutrients are needed to keep bones, teeth, and muscles healthy. A lack of vitamin D_3_ can lead to bone deformities such as rickets in young children and bone pain in adults resulting in osteoporosis, a condition where the bone weakens and becomes brittle.^2−5^ It has also been linked to various other diseases such as diabetes, heart disease, and neurological disorders such as Alzheimer’s disease and schizophrenia.^6−9^

**Figure 1:**
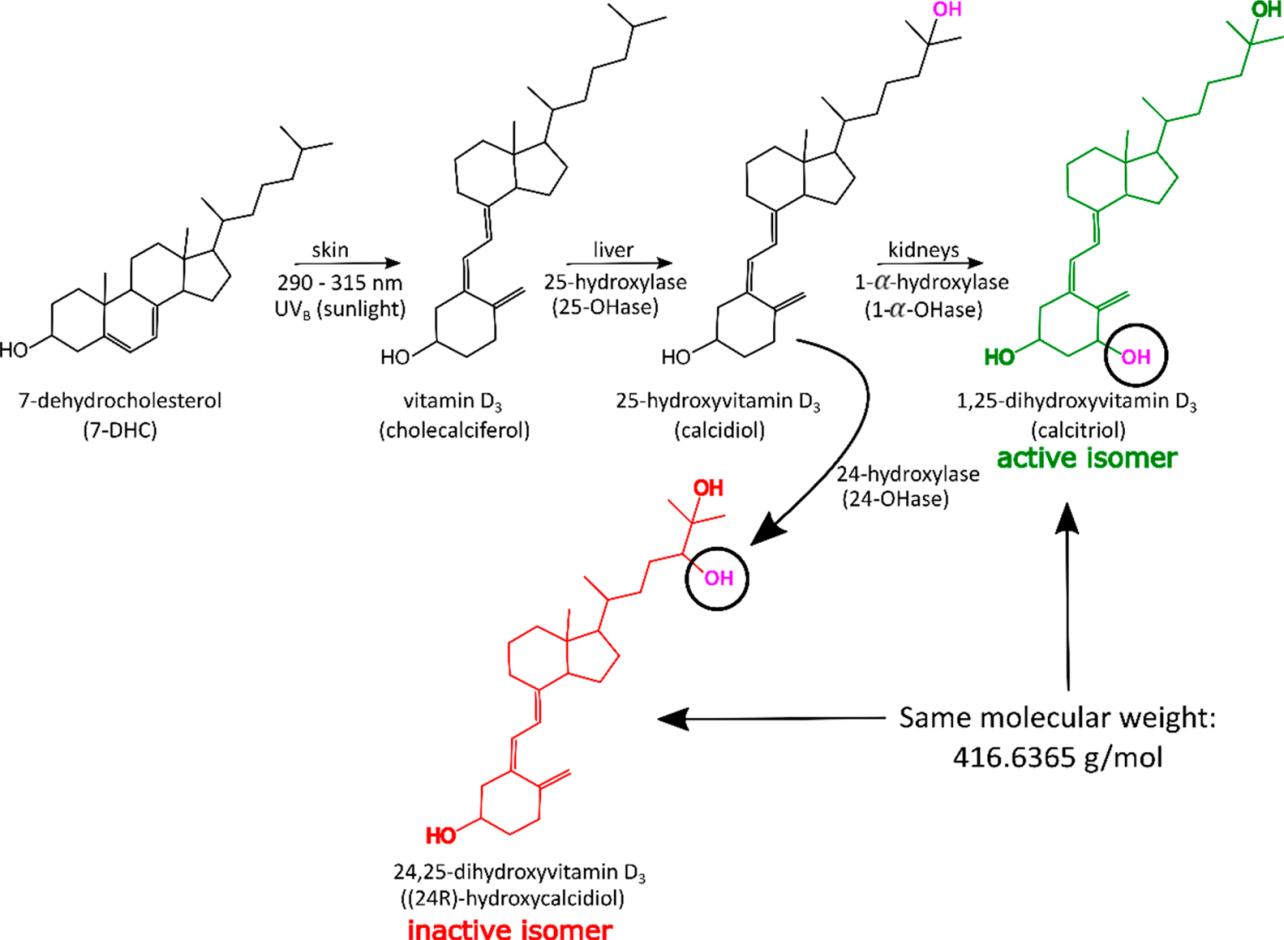
Pathway for vitamin D metabolism with the highlighted OH groups to emphasize the difference in structures of the dihydroxylated isomers. Redrawn and adapted from ref (25).

The metabolic pathway of vitamin D_3_ is illustrated by Figure 1. Vitamin D_3_ (cholecalciferol) is made in the skin from 7-dehydrocholesterol (7-DHC) under the influence of UV light (290–315 nm, UV_B_) from the sun.^1,10^ It metabolizes first to 25-hydroxyvitamin D_3_ (25(OH)D_3_) in the liver and is then further oxidized to the biologically active compound 1,25-dihydroxyvitamin D_3_ (1,25(OH)_2_D_3_).^10−17^ During catabolism of vitamin D_3_, 24,25-dihydroxyvitamin D_3_ is formed, which is usually considered to be inactive. However, there are some studies that show that this metabolite may have some biological activity of its own.^18−21^ For example, in 1982, Sömjen et al.^22^ found that 24,25(OH)_2_D_3_ may play a role in the metabolism of developing skeletal tissues of newborn mice, and Seo et al.^23^ showed that increased levels of 24,25(OH)_2_D_3_ levels in the serum may be correlated with the healing of tibial fractures in chicks.

The most abundant metabolite 25(OH)D_3_ is commonly used as a marker compound for vitamin D status because of its high concentration levels and because of its direct link to the vitamin D substrate. Using the active compound 1,25(OH)_2_D_3_ as a biomarker for vitamin D_3_ sufficiency is difficult as its half-life is only a few hours and its concentration levels are very low.^24^

Currently, immunoassays^26,27^ and liquid chromatography–tandem mass spectrometry (LC-MS/MS)^28,29^ are commonly used for detecting and determining the levels of vitamin D metabolites in humans. Immunoassays can take time as only one metabolite can be measured per assay so the selectivity, accuracy, and reproducibility may suffer as a result. LC–MS/MS assays, however, provide better selectivity, sensitivity, and reproducibility and are highly considered as one of the main techniques for the analysis of vitamin D metabolites, specifically including the separation of vitamin d isomers, which is usually carried out in the LC domain.^30−35^ However, due to the low abundance of certain metabolites such as 1,25(OH)_2_D_3_ and the complex matrices they are detected in such as human serum, qualitative and quantitative analyses can be difficult due to isobaric and isomeric interferences that can arise from biological fluids.^36^

Vitamin D metabolites have also been analyzed using gas chromatography–mass spectrometry (GC–MS), but the metabolites tend to require some modification or derivatization using agents such as trimethylsilyl (TMS).^37,38^ For LC–MS, derivatization reagents such as 4-phenyl-1,2,4-triazoline-3,5-dione (PTAD)^39−41^ or Amplifex^42^ have been used to improve the ionization efficiency of the vitamin D compounds and to also decrease isobaric interference levels coming from the media, e.g., serum by shifting the *m*/*z* range of the vitamin D metabolites to higher values.^43^ However, this adds an additional step to the sample preparation and may require the data to be interpreted more carefully.

Recently, MS methods have been further developed to differentiate between isomeric and epimeric vitamin D_3_ metabolites. For example, Qi et al.^44^ implemented a matrix-assisted laser desorption ionization–collision induced dissociation (MALDI–CID) method after ion activation of reactive analyte/matrix adducts to distinguish between dihydroxyvitamin D_3_ isomers (1,25(OH)_2_D_3_ and 24,25(OH)_2_D_3_).^44^ The CID MS/MS spectra of the reactive matrix (1,5 diaminonaphthalene)/dihydroxyvitamin D_3_ adducts formed during MALDI produced isomer-diagnostic fragment ions because the fragile OH groups were preserved during dissociation of the C-6/C-7 bond.^44^ As there were differences in the locations of the −OH groups, different product ions were obtained. Chouinard et al.^45^ tested the separation capabilities of ion mobility–mass spectrometry (IMS-MS) to distinguish between the gas-phase conformations of 25(OH)D_3_ epimers with the aid of theoretical modeling of the epimers.^45,46^ These developments have encouraged utilization of different mass spectrometry techniques to further characterize and elucidate the structures of vitamin D metabolites.

In this work, we investigated the use of a 12 T Fourier transform ion cyclotron resonance mass spectrometer (FT-ICR MS), equipped with various fragmentation methods to enable differentiation of the two dihydroxylated vitamin D_3_ isomers, without the need for prior chromatographic separation or derivatization of the samples. Slow heating fragmentation methods such as CID were revisited. Photodissociation methods such as IRMPD and UVPD MS/MS and electron mediated fragmentation techniques such as EID were also explored. Dehydrations were observed in the spectra using all methods and the fragments corresponding to the consecutive losses of the three water molecules were by far the most abundant. The MS/MS spectra were also equally dense due to the series of hydrocarbon chain decompositions. However, using all fragmentation methods, multiple diagnostic fragments were observed for the active metabolite, 1,25(OH)2D_3_, showing the retention of the fragile OH groups, whereas the characteristic fragments of 1,25(OH)_2_D_3_ were clearly absent for the 24,25(OH)_2_D_3_ isomer.

## Experimental Section

### Chemicals

Solvent-evaporated standards of 1,25(OH)_2_D_3_ (15 μg) and 24,25(OH)_2_D_3_ (10 μg) were provided by the Volmer group from Humboldt University of Berlin, Germany. Ultrapure water was obtained using a Millipore (Merck Millipore, MA) Direct-Q Milli-Q UV III purification system (18.2 Ω). LC–MS grade methanol (≥99.9%) was purchased from VWR Chemicals (Germany), and formic acid was purchased from Honeywell Fluka (Germany). The samples were prepared to stock solutions of 36 μM for dihydroxylated vitamin D_3_ isomers in methanol, which were then stored in the −80 °C freezer. Final samples were diluted with water/methanol (50:50, v/v) with 1% v/v formic acid into concentrations of 1–10 μM for MS, CID, IRMPD, EID, and UVPD MS/MS experiments.

### Mass Spectrometry

A 12 T (T) SolariX Fourier transform ion cyclotron resonance mass spectrometer (FTICR MS; Bruker Daltonik GmbH, Bremen, Germany) equipped with an actively shielded superconducting magnet was used for the experiments. Mass spectra were acquired with four mega data points (32 bit per point) over a mass range of *m*/*z* 98.2–1000 to produce a 1.12 s transient and ∼300,000 resolving power at *m*/*z* 400.

The samples were analyzed using a homemade nanoelectrospray ion source in positive ionization mode. Ions were externally accumulated in a hexapole collision cell for 0.5 s before they were transferred to the ICR analyzer cell for MS detection.

For all MS/MS experiments, the protonated molecules were isolated using the quadrupole mass filter with an isolation window of 5 *m*/*z*. For CID MS/MS after mass isolation of the precursor ion, argon was used as the collision gas and the resulting fragments were accumulated in the collision cell. The collision energy was optimized to 10 V.

A 25 W continuous wave CO_2_ laser (Synrad, Mukilteo, WA) was employed for the IRMPD MS/MS experiments with an output wavelength of 10.6 μm, pulse length of 0.1 s, and 50% laser power.

For the EID MS/MS experiments, the quadrupole isolated ions were accumulated in the hexapole for 1 s. Isolated ions of interest were transferred and trapped in the ICR cell. The trapped ions were then irradiated by electrons from a 1.5 A indirectly heated hollow dispenser cathode. The EID parameters used were a pulse length of 0.4 V, cathode bias of 19 V and extraction lens voltage of 3 V.

For the ultraviolet photodissociation experiments (UVPD), a 193 nm ArF excimer laser beam (10 Hz, Coherent, UK) was introduced into the back of the ICR cell of the instrument through a BaF_2_ window, and ions stored in the cell were irradiated with five laser shots (5 mJ/pulse at the laser head). No hardware modifications were required due to the pre-existing IRMPD setup which allowed simple alignment of the UV laser.

A stable telescopic compact high energy Q-switched pulsed Nd:YAG laser with an output wavelength of 213 nm (fifth harmonic of the Nd:YAG laser) (10 Hz; Litron Lasers, UK) was also used for UVPD, and ions were irradiated with 10 laser shots (∼1.5 mJ/pulse at the laser head).

All spectra were internally calibrated, manually interpreted and assigned via DataAnalysis 4.3 software (Bruker Daltonik, GmbH, Bremen, Germany) to achieve subppm accuracy for all assigned fragments in the MS and MS/MS spectra.

## Results and Discussion

**Figure 2:**
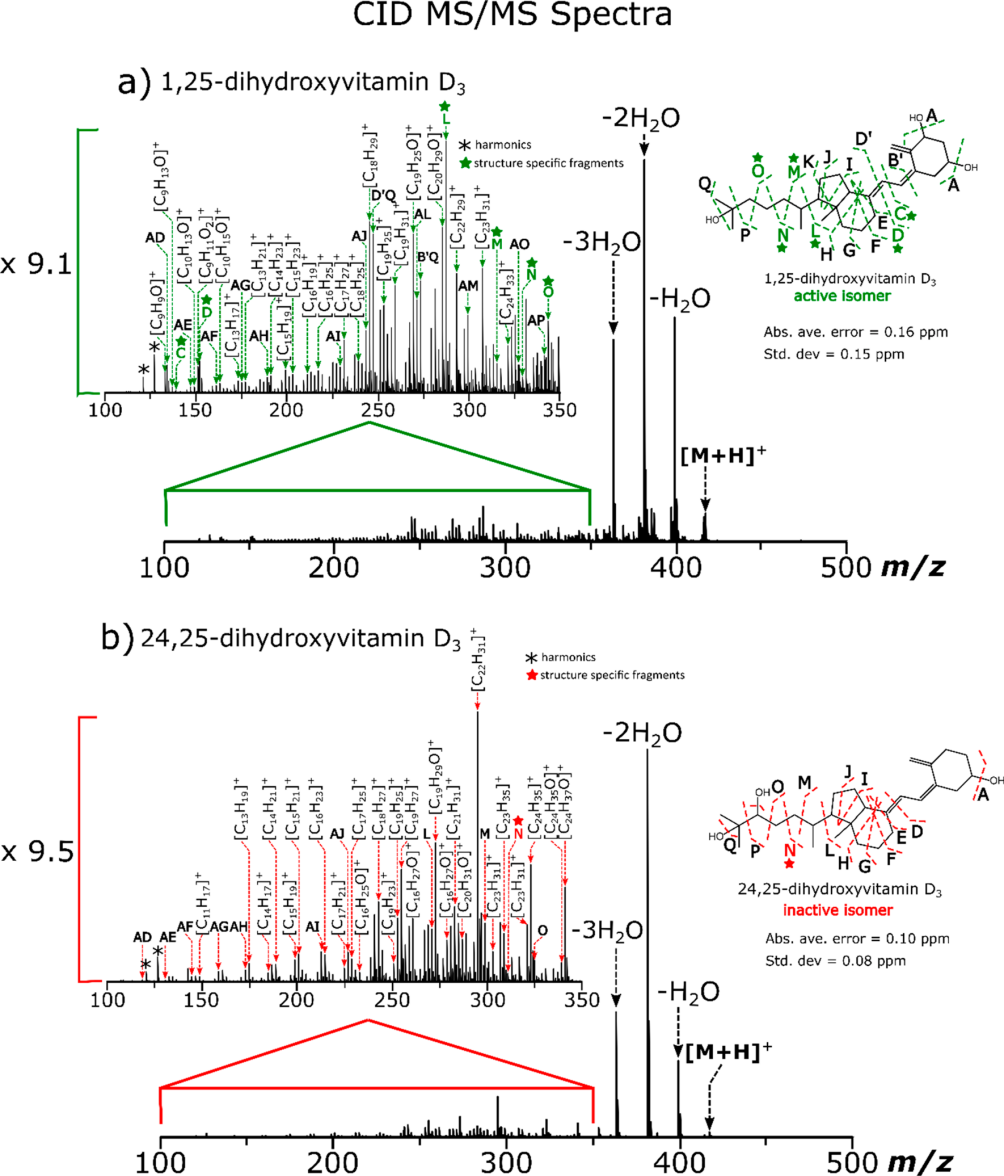
CID spectra with inserts of *m*/*z* 100–350 regions with fragment peaks labeled of (a) 1,25(OH)_2_D_3_ and (b) 24,25(OH)_2_D_3_. Structure-specific fragments are denoted by the star symbol.

Full MS analysis of the dihydroxylated vitamin D_3_ isomers showed that the protonated molecule for both isomers was present at the same *m*/*z*, demonstrating that it is not possible to differentiate between the isomers simply based on the mass spectra (Figure S1). As illustrated in Figure 2, three major peaks were clearly identified in all of the MS/MS spectra, corresponding to initial dehydrations (loss of H_2_O). This was also observed in the MS confirming the fragile nature of the OH groups of the isomeric species. Schorr et al. have recently shown that H_2_O loss from the protonated OH group at C-3 or C-25 only requires activation energies of 10–15 kcal·mol^–1^.^47^ A homologous series of hydrocarbon losses (−CH_2_) resulting from direct carbon–carbon (C–C) cleavages were also observed in all the fragmentation spectra. These fragments contribute to the complex spectra and provide no structural information or any isomer-specific fragments for the species analyzed.

In previous studies, the use of CID for vitamin D_3_ compounds resulted in complex spectra often accompanied by limited structural information.^36,48^ Some of the other issues noted for these fat-soluble compounds including the lack of ionizable groups as well as analysis of these metabolites in complex matrices such as serum can be difficult, as the compounds are already present in low levels and interference from other species present in the matrices can contribute to the ion suppression for the vitamin D_3_ compounds such as 1,25(OH)_2_D_3_. Hence, these experiments are primarily tested on the provided standards of the VD_3_ isomers as a basis for method development for differentiation of the isomers using the available MS/MS methods.

As mentioned for the CID MS/MS spectra shown in Figure 2, the same observations can be made for the IRMPD, EID, and UVPD MS/MS spectra obtained (Figures S3–S6), which also present the significant water losses and the series of intense fragment peaks resulting from the hydrocarbon (−CH_2_) losses.

**Figure 3:**
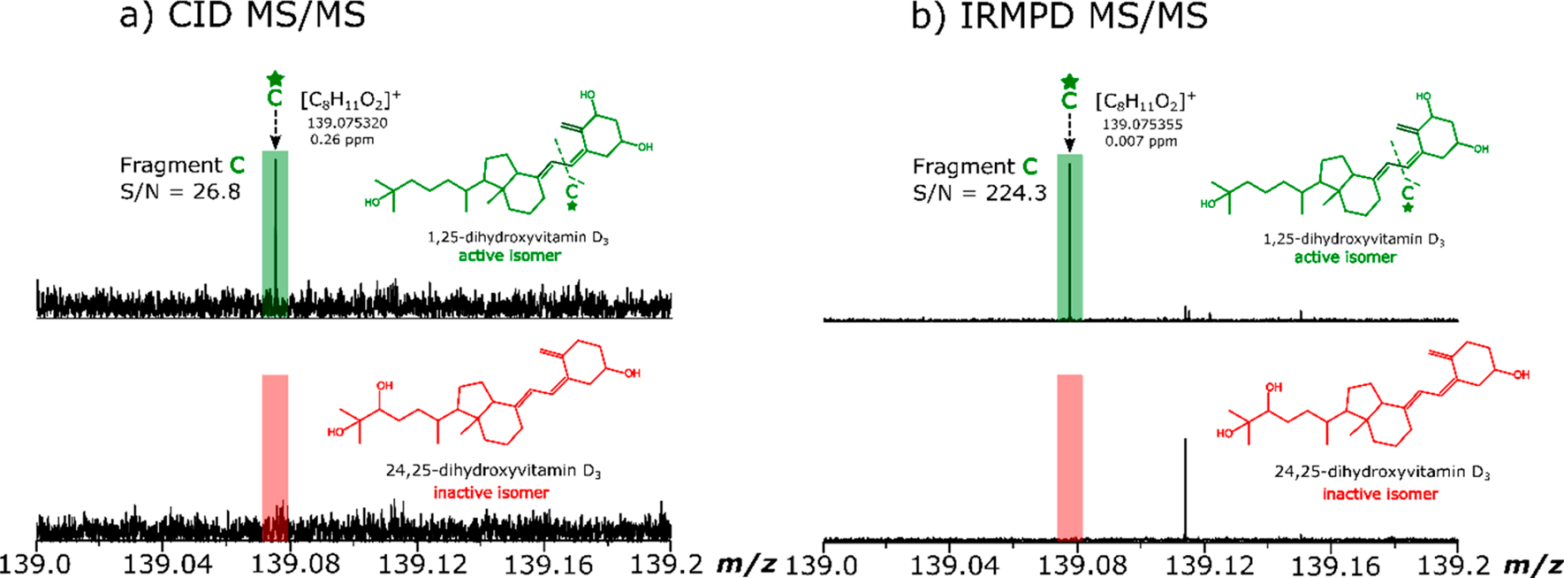
Zoom in of *m*/*z* 139.0–139.2 region of (a) CID MS/MS spectra and (b) IRMPD MS/MS spectra of 1,25(OH)_2_D_3_ and 24,25(OH)_2_D_3_ showing 1,25(OH)_2_D_3_-specific fragment C. An 8-fold improvement in the S/N is also noted for the diagnostic fragment C using IRMPD MS/MS compared to CID MS/MS.

After collision energy optimization and on closer inspection and analysis of the MS/MS spectra, diagnostic fragments were detected for 1,25(OH)_2_D_3_, which were absent in the 24,25(OH)_2_D_3_ MS/MS spectra. An example of this is shown in Figure 3, observed for both CID and IRMPD (also observed with the other MS/MS methods), where the detected fragment at *m*/*z* 139.07 in the spectrum for 1,25(OH)_2_D_3_ was present, while it was absent in the 24,25(OH)_2_D_3_ MS/MS spectrum. This indicates that the fragile OH groups can be preserved during dissociation for one isomer but not for the other. This may be due to a difference in the energetics between both isomers as 24,25(OH)_2_D_3_; however, this is under strong consideration as the structural difference between both isomers is minor since the only difference is that 24,25(OH)_2_D_3_ has only one OH group on the A ring whereas 1,25(OH)_2_D_3_ has two OH groups on the A ring. On the other hand, Schorr et al.^47^ have recently demonstrated significant structural and energetic differences between the 25(OH)D_3_ epimers, which only differ in the stereochemical orientation of the C-3 hydroxyl group, due to differences in intramolecular H-bonding.

It is also noted that the signal of the protonated molecule in the 1,25(OH)_2_D_3_ CID MS/MS spectra is higher in intensity and, thus more stable compared to the signal of the protonated molecule in the 24,25(OH)_2_D_3_ CID MS/MS spectra (Figure 2) as well as the IRMPD, UVPD, and EID spectra (Figures S3–S6). This observation may also provide some insight as to why the characteristic fragments obtained for 1,25(OH)_2_D_3_ demonstrate the retention of either one or both OH groups on the compounds, which was not possible for the 24,25(OH)_2_D_3_ isomer.

**Figure 4:**
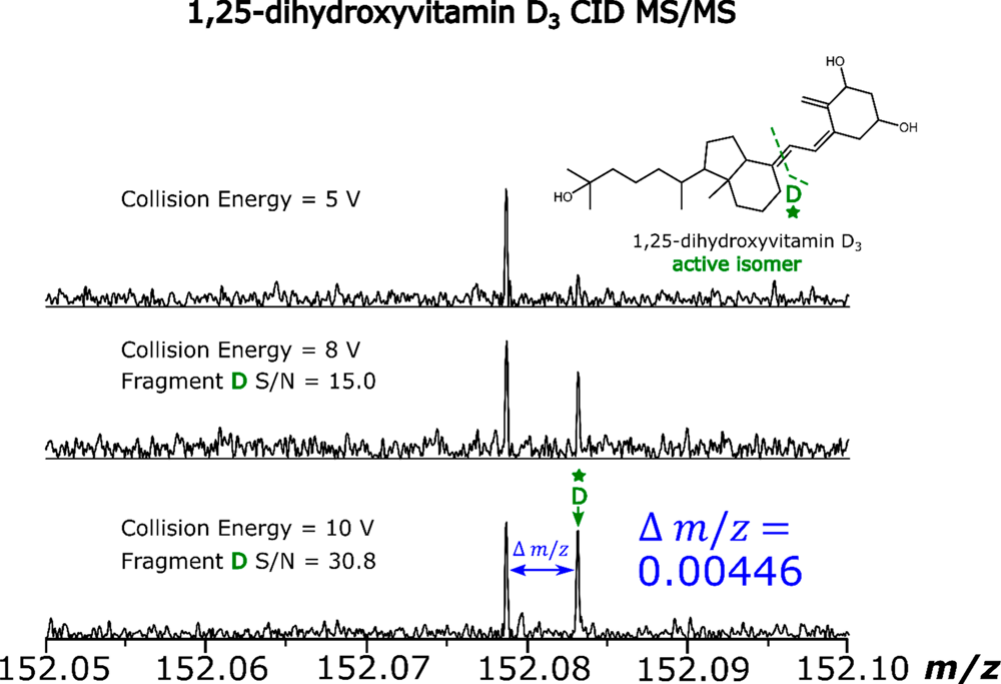
*m*/*z* scale expansions of the region *m*/*z* 152.05–152.10 from the CID MS/MS spectra of 1,25-dihydroxyvitamin D_3_ after collision energy optimization.

For each MS/MS method, the parameters required for fragmentation optimization were tuned, and up to 100 scans were accumulated to ensure that the characteristic fragments observed for the 1,25(OH)_2_D_3_ spectra were absent for 24,25(OH)_2_D_3_ spectra. This included optimization of the collision energy for CID MS/MS experiments, the pulse length for ion interaction with IR or UV photons for both IRMPD and UVPD MS/MS, as well as the bias voltage, which is responsible for the energy of the electrons for the EID MS/MS experiments. Figure 4 shows how the optimization of the collision energy was necessary for the detection of one of the characteristic fragments of 1,25(OH)_2_D_3_, which appeared to be absent when a collision energy of 5 V was applied but present when the optimized collision energy of 10 V was used.

**Figure 5:**
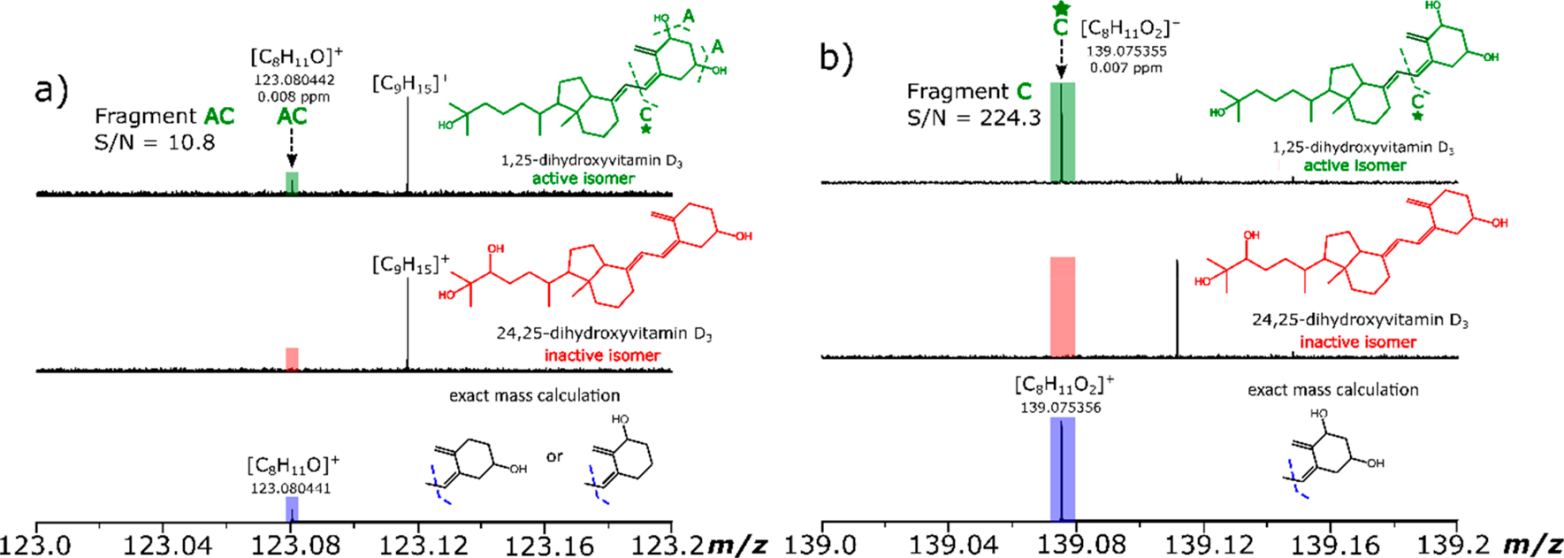
*m*/*z* scale expansions of IRMPD spectra obtained of (a) *m*/*z* 123.0–123.2 and (b) *m*/*z* 139.0–142.2 for the IRMPD fragment ions of 1,25(OH)_2_D_3_ (top traces) and 24,25(OH)_2_D_3_ (middle traces). The exact mass calculation and simulation of the assigned elemental formulas with chemical structures are shown in the bottom trace.

The internal calibration of all fragmentation spectra (included in this work and in the Supporting Information) resulted in subppm mass accuracy assignment errors. Assignments were made with high confidence as the following criteria were followed closely. For example, all product ions and, in particular, the characteristic fragments for the differentiation between the isomers, were checked manually, based on low mass errors (<1 ppm) as well ensuring that the exact mass calculation and simulation of each characteristic fragment matched with the observed fragment in the MS/MS spectra obtained. It is important to have subppm mass assignments for the fragments as multiple assignments are possible; hence, it is also necessary to accompany this with the exact mass calculation and simulation of the assigned elemental formulas as shown in Figure 5a,b.

Multiple diagnostic fragments for 1,25(OH)_2_D_3_ were detected as shown in Table 1. The table displays the main characteristic fragments detected in the 1,25(OH)_2_D_3_ spectra, which were definitively absent in the 24,25(OH)_2_D_3_ MS/MS spectra using the various MS/MS methods available. This was shown only for the 1,25(OH)_2_D_3_ isomer as this metabolite had fragments that were also generated for 24,25(OH)_2_D_3_ due to the fragile OH groups on the A ring and the side chain of the molecule. The assignment of the diagnostic fragments corresponds to the assigned cleavages of the 1,25(OH)_2_D_3_ compound; e.g., fragment “AD” refers to bonds “A” and “D” broken in the 1,25(OH)_2_D_3_ compound, as shown by the cleavage diagram in Figure 2.

**Table 1:** Fragmentation Table for Characteristic Fragments, Where One or Both OH Groups Are Retained on the Ring for 1,25(OH)_2_D_3_ and Are Absent in the 24,25(OH)_2_D_3_ Spectra^a^

		fragmentation method									
		CAD		IRMPD		EID		193 nm UVPD		213 nm UVPD	
1,25(OH)2D3 characteristic theoretical fragment(*m*/*z*)	assignment	intensity	S/N	intensity	S/N	intensity	S/N	intensity	S/N	intensity	S/N
109.064791	AB	X	X	X	X	medium	18.1	medium	53.6	medium	58
127.075356	B	X	X	high	231.5	medium	18.9	low	23.1	low	42
135.080441	AD	high	346.4	high	639.4	high	106.7	high	585.7	high	507
139.075356	C	low	26.8	high	224.3	medium	12.6	low	29.5	low	27.1
147.080441	AE	low	20	low	27.7	low	21.2	medium	40.9	low	40.6
152.083181	D	low	30.8	medium	63.6	high	42.2	medium	36.9	low	24.7
165.091006	E	low	44.2	medium	70.2	low	12.8	low	14.7	low	12.2
287.200557	L	high	390.1	high	423.9	medium	198.7	high	141.2	low	25.3
315.231857	M	high	27.2	medium	77.6	low	39.6	medium	38.3	low	9.2
329.247507	N	low	128.1	low	26.5	low	12.1	low	15.8	X	X
343.263157	O	high	493.1	medium	67.6	low	53.3	medium	36.9	X	X
357.278807	P	medium	78	low	16.7	X	X	X	X	X	X

a In the table, “X” denotes the absence of the fragment in the 1,25(OH)_2_D_3_ MS/MS spectra, and further explanation about the fragment intensity level is provided in Table 2.

Testing all of the available fragmentation methods presents an opportunity for comparison of the suitability of each method for qualitative and quantitative analysis. Depending on the MS/MS method used, the metabolites may undergo a different fragmentation pathway, resulting in secondary fragmentation, improvement in the number of diagnostic fragments detected, or an improvement in the relative intensities of those diagnostic fragments. This is summarized in Table 1, and the relative intensity range used to designate the fragment intensity levels for the characteristic fragments of 1,25(OH)_2_D_3_ is provided in Table 2.

**Table 2:** Table Showing the Relative Intensity Range Used to Designate the Fragment Intensity Levels for the Characteristic Fragments of 1,25(OH)_2_D_3_

fragment intensity level	intensity range
low	1 × 10^6^ – 5 × 10^6^
medium	5 × 10^6^ – 1 × 10^7^
high	>1 × 10^7^

As shown by Table 1, the same main characteristic fragments (except for fragment B) were observed in the CID and IRMPD MS/MS spectra of 1,25(OH)_2_D_3_, but an improvement in the intensities and S/N of those same fragments was also observed with IRMPD MS/MS. With EID, however, the relative intensity of the diagnostic and nondiagnostic fragments was overall lower compared to both CID and IRMPD, yet complementary structural information was obtained with EID and an additional diagnostic fragment at *m*/*z* 109.06 (AB) was also observed. EID uses higher energy electrons and is a radical-based process, and these reasons may contribute to complexity of the EID spectra obtained and the presence of the additional fragment observed.

Compared to IRMPD, UVPD is a higher energy activation method based on the absorption of UV photons by the analyte ions, which is possible due to the UV chromophore properties of the C–C double bonds present in the 5,6-cis-triene system of the vitamin D compounds. The structural information obtained with 193 nm UVPD for the dihydroxylated vitamin D_3_ compounds also compared well with the MS/MS data obtained with CID, IRMPD, and EID MS/MS. This observation may be supported by a combination of the previously proposed UVPD mechanisms; direct dissociation (electronic excitation or relaxation into a dissociative orbital, like that of electron-based fragmentation methods e.g., EID) and internal conversion (internal conversion of the photon energy into vibrational modes results in fragmentation in the ground state so the fragments generated will be like those generated by CID and IRMPD).^49−51^

With 213 nm UVPD, the fragments obtained were low intensity compared to other MS/MS methods, yet structure-specific fragments and cross-ring cleavages across both molecules were observed. It is difficult to make a direct comparison between the performance of the 193 and 213 nm UVPD on the data obtained as the number of laser shots and the energy output for each laser were different. However, as shown in Table 1, although most fragments were low intensity, many of the isomer-specific fragments (nine out of the 12) listed for 1,25(OH)_2_D_3_ were detected with 213 nm UVPD MS/MS.

**Figure 6:**
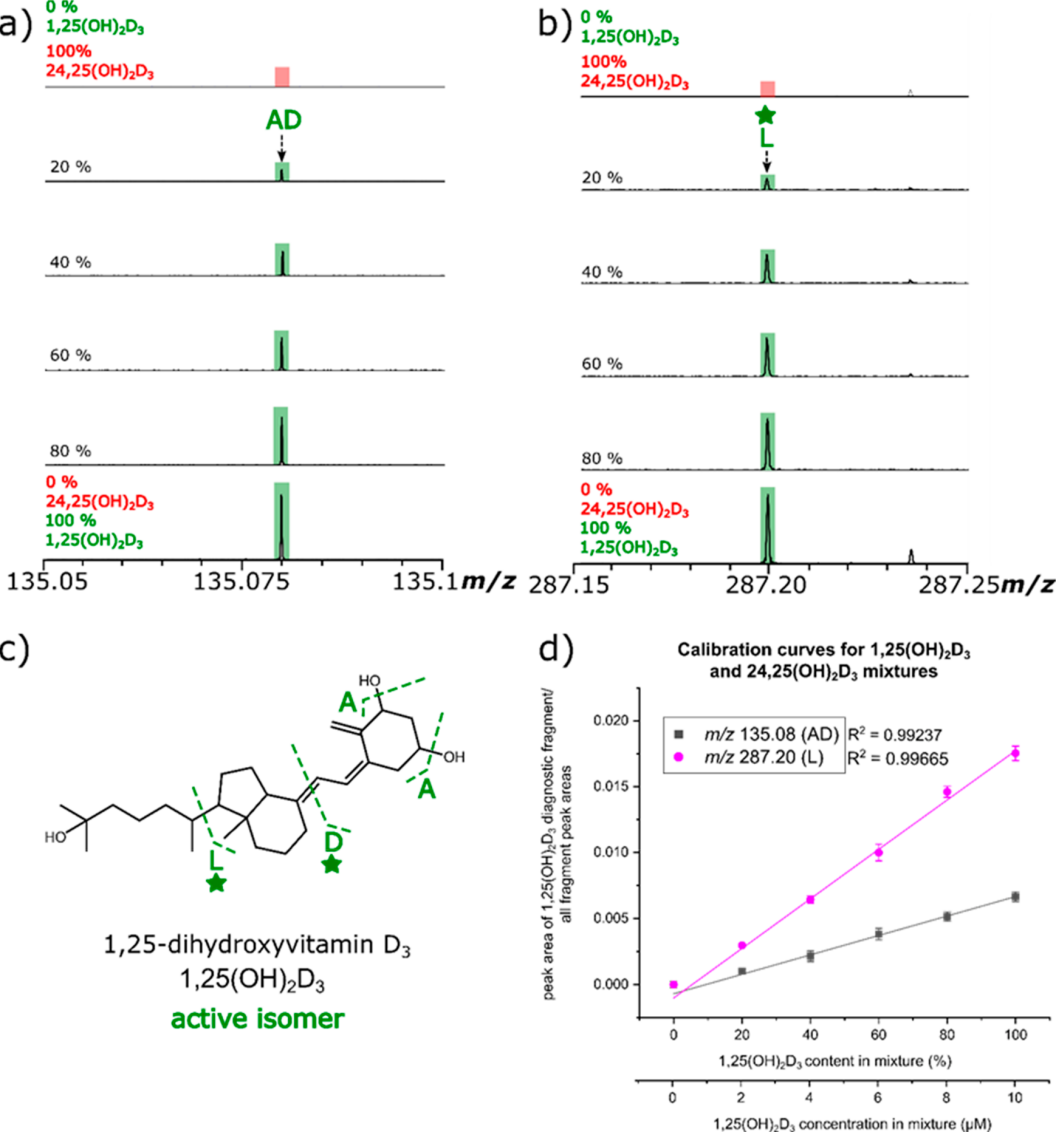
*m*/*z* scale expansion of (a) *m*/*z* 135.05–135.10 and (b) *m*/*z* 287.15–287.25 from the IRMPD spectra for the characteristic 1,25(OH)_2_D_3_ IRMPD fragment ions with increasing increments of 1,25(OH)_2_D_3_ in percentage concentration in the mixture. (c) Structure of 1,25(OH)_2_D_3_ with associated cleavages to produce the diagnostic fragments “AD” and “L”. (d) Calibration curves generated using the peak area ratio of the 1,25(OH)_2_D_3_-specific “AD” and “L” fragments.

A direct infusion relative quantification method is discussed herein using the dihydroxylated vitamin D standards. The highlighted 1,25(OH)_2_D_3_-specific product ions *m*/*z* 135.08 and 287.20 were chosen to test if the relative quantitation of the isomers was possible as these fragments had the highest relative intensities and S/N out of the characteristic fragments listed in Table 1. Mixtures of 1,25(OH)_2_D_3_ and 24,25(OH)_2_D_3_ were prepared at known concentration ratios in which the 1,25(OH)_2_D_3_ content varied from 0 to 100% in 20% increments. Parts a and b of Figure 6 demonstrate that it is possible to discriminate between the dihydroxylated vitamin D_3_ isomers and show that, as the percentage of 1,25(OH)_2_D_3_ in the 1,25(OH)_2_D_3_ /24,25(OH)_2_D_3_ standard mixtures is increased the intensity of the IRMPD fragments at *m*/*z* 135.08 and 287.2 also increased in intensity.

A ratio was taken of the peak area of the 1,25(OH)_2_D_3_ specific fragment to the sum of all the fragments present in the IRMPD MS/MS spectrum for each isomer mixture. Fluctuations were observed in the calibration curve when only the peak area or the peak intensities of the characteristic fragment were plotted against the percentage of 1,25(OH)_2_D_3_ in the dihydroxylated vitamin D_3_ isomeric mixture. Calibration curves were obtained with good linearity (*R*^2^ > 0.99) with the inclusion of the confidently assigned (mass error <1 ppm) fragments and using the equation below:
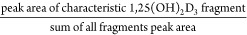


## Conclusions

In this study, the use of CID was revisited and alternative fragmentation methods such as IRMPD, UVPD, and EID MSMS/MS were investigated for the differentiation of the isomeric dihydroxylated vitamin D_3_ compounds. Extensive fragmentation including cross-ring cleavage of both dihydroxylated VD_3_ isomers was observed with all fragmentation methods applied. More significantly, isomer-specific fragments were observed for 1,25-dihydroxyvitamin D_3_, which were absent for 24,25-dihydroxyvitamin D_3_ after optimization of the parameters for each MS/MS method and accumulation of scans. The structure-specific fragments generated due to cleavage of the C-6/C-7 bond in the 1,25-dihydroxyvitamin D_3_ compound demonstrate that the OH groups were retained during dissociation using all the available fragmentation methods.

It should be noted that the water losses and series of hydrocarbon losses for both isomers dominate all the MS/MS spectra obtained. However, after detailed analysis, multiple characteristic fragments were found with the aid of the high resolving power and mass accuracy provided by the FT-ICR MS, which was fully equipped with all the different MS/MS methods.

In summary, diagnostic fragments were observed for 1,25-dihydroxyvitamin D_3_, enabling quick and easy differentiation between the two dihydroxylated vitamin D_3_ isomers without the need for prior chromatographic separation or derivatization of the molecules. Preliminary experiments for the quantitative analysis of 1,25-dihydroxyvitamin D_3_ were carried out, and a linear calibration curve using the diagnostic fragments observed for 1,25-dihydroxyvitamin D_3_ was established (*R*^2^ > 0.99).

This direct infusion quantification method using MS/MS has the potential to be applied to the vitamin D_3_ metabolites detected in matrices such as serum, which are routinely found in low concentrations and often masked by other endogenous material; hence, chromatographic separation prior to MS/MS analysis may be beneficial while the characteristic fragments listed in this work can be used to identify and quantify the biologically active 1,25-dihydroxyvitamin D_3_ compound.

## Supporting Information Available

Tables of peak assignments of all spectra, as well as figures of mass spectra, mass isolation spectra, and tandem mass spectra (IRMPD, 193 nm UVPD, 213 nm UVPD, and EID) of 1,25 dihydroxylated vitamin D_3_ and 24,25 dihydroxylated vitamin D_3_; equation used to calculate the percentage fragmentation intensity to precursor intensity ratio of the characteristic fragments for 1,25-dihydroxyvitamin D_3_ (PDF)
