# Supplementary material for: Differentiation of Dihydroxylated Vitamin D3 Isomers Using Tandem Mass Spectrometry
Source: J Am Soc Mass Spectrom. 2022 May 13;33(6):1022–30. doi: 10.1021/jasms.2c00085 (PMC9164238; doi:10.1021/jasms.2c00085)
Supplement: Supplementary file 1 — js2c00085_si_001.pdf [file js2c00085_si_001.pdf]

Supporting Information

Differentiation of dihydroxylated vitamin D_3 isomers using tandem mass spectrometry – supporting information

Anisha Haris[1], Yuko P. Y. Lam[1], Christopher A. Wootton[1], Alina Theisen[1], Bryan P. Marzullo[1], Pascal Schorr[2], Dietrich Volmer[2], and Peter B. O'Connor*[1]

[1]Department of Chemistry, University of Warwick, Coventry, CV4 7AL, UK
[2]Institut für Chemie, Humboldt-Universität zu Berlin, 12489 Berlin, Germany

*Corresponding author email address: p.oconnor@warwick.ac.uk

Contents:

Table S 1: Peak assignment table for the CID MS/MS of the protonated 1,25 dihydroxylated vitamin D_3 isomer.*Represents the peaks that were used for internal calibration. 2
Table S 2: Peak assignment table for the CID MS/MS of the protonated 24,25 dihydroxylated vitamin D_3 isomer. *Represents the peaks that were used for internal calibration. 5
Table S 3: Peak assignment table for the IRMPD MS/MS of the protonated 1,25 dihydroxylated vitamin D_3 isomer. *Represents the peaks that were used for internal calibration. 7
Table S 4: Peak assignment table for the IRMPD MS/MS of the protonated 24,25 dihydroxylated vitamin D_3 isomer. *Represents the peaks that were used for internal calibration. 10
Table S 5: Peak assignment table for the 193 nm UVPD MS/MS of the protonated 1,25 dihydroxylated vitamin D_3 isomer. *Represents the peaks that were used for internal calibration. 12
Table S 6: Peak assignment table for the 193 nm UVPD MS/MS of the protonated 24,25 dihydroxylated vitamin D_3 isomer. *Represents the peaks that were used for internal calibration. 15
Table S 7: Peak assignment table for the 213 nm UVPD MS/MS of the protonated 1,25 dihydroxylated vitamin D_3 isomer. *Represents the peaks that were used for internal calibration. 17
Table S 8: Peak assignment table for the 213 nm UVPD MS/MS of the protonated 24,25 dihydroxylated vitamin D_3 isomer. *Represents the peaks that were used for internal calibration. 19
Table S 9: Peak assignment table for the EID MS/MS of the protonated 1,25 dihydroxylated vitamin D_3 isomer. *Represents the peaks that were used for internal calibration. 21
Table S 10: Peak assignment table for the EID MS/MS of the protonated 24,25 dihydroxylated vitamin D_3 isomer. *Represents the peaks that were used for internal calibration. 23
Table S 11: Fragmentation table for characteristic fragments, where one or both OH groups retained on the ring for $1,25(OH)_2D_3$ which are absent in the $24,25(OH)_2D_3$ MS/MS spectra. 25

Figure S 1: Mass spectrum of a) 1,25-dihydroxylated vitamin D_3 and b) 24,25-dihydroxylated vitamin D_3. 26
Figure S 2: Mass isolation spectrum with an *m/z* isolation window of 5 *m/z* for a) 1,25 dihydroxylated vitamin D_3 and b) 24,25 dihydroxylated vitamin D_3. 27
Figure S 3: IRMPD MS/MS spectra with inserts of *m/z* 100 – 350 regions with fragment peaks labelled for a) 1,25-dihydroxyvitamin D_3 and b) 24,25-dihydroxyvitamin D_3. 28
Figure S 4: 193 nm UVPD MS/MS spectra with inserts of *m/z* 100 – 350 regions with the fragment peaks labelled for a) 1,25-dihydroxyvitamin D_3 and b) 24,25-dihydroxyvitamin D_3. 29
Figure S 5: 213 nm UVPD MS/MS spectra with inserts of *m/z* 100 – 350 regions with the fragment peaks labelled for a) 1,25-dihydroxyvitamin D_3 and b) 24,25-dihydroxyvitamin D_3. 30
Figure S 6: EID MS/MS spectra with inserts of *m/z* 100 – 350 regions with fragment peaks labelled for a) 1,25-dihydroxyvitamin D_3 and b) 24,25-dihydroxyvitamin D_3. 31

Equation S 1: Equation to calculate the percentage fragmentation intensity to precursor intensity ratio for the characteristic fragments for 1,25-dihydroxyvitamin D_3. 31

Table S 1: Peak assignment table for the CID MS/MS of the protonated 1,25 dihydroxylated vitamin D_3 isomer.*Represents the peaks that were used for internal calibration.

Assignment	Elemental composition	Intensity	Resolution	Theoretical *m/z*	Observed *m/z*	Mass error (ppm)
	C_9H_{11}	2061866	1077261	119.085527	119.085441	-0.72
	C_9H_9O	5472289	947214	133.064791	133.064727	-0.48
AD	$C_9H_{11}O$	23938552	920511	135.080441	135.080393	-0.36
	$C_9H_{13}O$	15070955	912936	137.096091	137.096044	-0.34
C	$C_8H_{11}O_2$	1983347	950057	139.075356	139.07532	-0.26
	$C_{11}H_{13}$	5323683	868950	145.101177	145.10113	-0.32
AE	$C_{10}H_{11}O$	1526401	949151	147.080441	147.080399	-0.29
	$C_{10}H_{13}O$	9244278	824438	149.096091	149.096066	-0.17
	$C_9H_{11}O_2$	25602036	833988	151.075356	151.075332	-0.16
	$C_{10}H_{15}O$	2383356	878831	151.111742	151.111715	-0.18
D	$C_9H_{12}O_2$	2293053	825058	152.083181	152.083144	-0.24
	$C_9H_{13}O_2$	22569352	820295	153.091006	153.090978	-0.18
	$C_{12}H_{15}$	9021058	803599	159.116827	159.116812	-0.09
AF	$C_{11}H_{13}O$	3294005	801681	161.096091	161.096086	-0.03
	$C_{11}H_{15}O$	2908662	829572	163.111742	163.111731	-0.07
E	$C_{10}H_{12}O_2$	3280050	774403	165.091006	165.090998	-0.04
	$C_{13}H_{15}$	10664191	749222	171.116827	171.116816	-0.06
	$C_{13}H_{17}$	12257985	716753	173.132477	173.132466	-0.06
AG	$C_{12}H_{15}O$	4327030	729898	175.111742	175.111746	0.02
	$C_{13}H_{19}$	11398270	722067	175.148127	175.148116	-0.06
	$C_{13}H_{21}$	13291060	715052	177.163777	177.163786	0.05
	$C_{13}H_{23}$	9741283	695438	179.179427	179.179421	-0.03
	$C_{14}H_{17}$	15953049	681392	185.132477	185.132492	0.08
*	$C_{14}H_{19}$	13559317	672381	187.148127	187.148127	0.00
AH	$C_{13}H_{17}O$	9007994	670989	189.127392	189.127389	-0.02
	$C_{14}H_{21}$	20266880	670093	189.163777	189.163776	-0.01
	$C_{14}H_{23}$	22655194	652927	191.179427	191.179424	-0.02
	$C_{12}H_{17}O_2$	2021912	643289	193.122306	193.122326	0.10
	$C_{14}H_{25}$	6070819	667615	193.195077	193.195111	0.18
	$C_{15}H_{17}$	13907062	653713	197.132477	197.132507	0.15
	$C_{15}H_{19}$	26645904	632977	199.148127	199.148148	0.11
	$C_{15}H_{21}$	16617116	623389	201.163777	201.163789	0.06
	$C_{15}H_{23}$	21587866	621190	203.179427	203.179429	0.01
CO	$C_{15}H_{25}$	9506955	611478	205.195077	205.195091	0.07
	$C_{16}H_{17}$	11917886	609393	209.132477	209.132477	0.00
	$C_{16}H_{19}$	19204876	594058	211.148127	211.148169	0.20
	$C_{16}H_{21}$	25816014	592531	213.163777	213.163809	0.15
	$C_{16}H_{23}$	19586184	584901	215.179427	215.17945	0.11
	$C_{16}H_{25}$	27176248	579544	217.195077	217.195081	0.02
CP	$C_{16}H_{27}$	23883234	574684	219.210727	219.210734	0.03
	$C_{16}H_{19}O$	33476658	552356	227.143042	227.143073	0.14
	$C_{17}H_{23}$	34156596	552632	227.179427	227.179442	0.07

Assignment	Elemental composition	Intensity	Resolution	Theoretical *m/z*	Observed *m/z*	Mass error (ppm)
AI	$C_{16}H_{21}O$	17477818	544501	229.158692	229.158702	0.04
	$C_{17}H_{25}$	24039612	542390	229.195077	229.195124	0.21
	$C_{16}H_{23}O$	15388990	537950	231.174342	231.174342	0.00
AJ	$C_{17}H_{23}O$	26235928	518740	243.174342	243.174335	-0.03
	$C_{18}H_{27}$	81663000	515083	243.210727	243.210792	0.27
	$C_{18}H_{29}$	201938064	509890	245.226377	245.226437	0.24
I	$C_{16}H_{23}O_2$	19768582	504654	247.169256	247.169277	0.08
D'Q	$C_{18}H_{31}$	205870352	507123	247.242027	247.242063	0.15
*	$C_{19}H_{27}$	55498488	493964	255.210727	255.210728	0.00
	$C_{19}H_{29}$	75192184	488940	257.226377	257.226408	0.12
	$C_{19}H_{31}$	147085312	484034	259.242027	259.242058	0.12
J	$C_{17}H_{25}O_2$	12384383	480345	261.184906	261.184876	-0.11
	$C_{18}H_{29}O$	6879361	470525	261.221292	261.221314	0.08
	$C_{18}H_{31}O$	9625863	469322	263.236942	263.236988	0.17
	$C_{19}H_{23}O$	5939731	420864	267.174342	267.17431	-0.12
	$C_{19}H_{25}O$	166222496	466510	269.189992	269.19006	0.25
AL	$C_{19}H_{27}O$	97180456	463452	271.205642	271.205654	0.04
*B'Q	$C_{20}H_{33}$	84089784	458038	273.257677	273.257724	0.17
	$C_{18}H_{27}O_2$	11697216	450225	275.200557	275.200592	0.13
	$C_{21}H_{29}$	115039728	447404	281.226377	281.226436	0.21
	$C_{21}H_{31}$	46259840	443816	283.242027	283.24206	0.12
	$C_{20}H_{29}O$	48783116	442013	285.221292	285.221289	-0.01
L	$C_{19}H_{27}O_2$	332488608	436153	287.200557	287.20064	0.29
	$C_{19}H_{29}O_2$	36806704	434029	289.216207	289.216236	0.10
	$C_{22}H_{29}$	52945236	429591	293.226377	293.226361	-0.05
*	$C_{22}H_{31}$	16298472	425753	295.242027	295.242021	-0.02
	$C_{22}H_{33}$	18879100	418298	297.257677	297.257696	0.06
AM	$C_{21}H_{31}O$	84763408	420850	299.236942	299.236906	-0.12
	$C_{22}H_{35}$	14971663	406694	299.273328	299.273275	-0.18
	$C_{23}H_{31}$	144401744	408611	307.242027	307.242037	0.03
	$C_{23}H_{33}$	42034664	407296	309.257677	309.257699	0.07
	$C_{23}H_{35}$	13117050	389140	311.273328	311.273278	-0.16
AN	$C_{22}H_{33}O$	4190985	394200	313.252592	313.252551	-0.13
M	$C_{21}H_{31}O_2$	26888088	399910	315.231857	315.231733	-0.39
	$C_{24}H_{35}$	69429728	390699	323.273328	323.273317	-0.03
	$C_{24}H_{37}$	21056110	378065	325.288978	325.288971	-0.02
AO	$C_{23}H_{35}O$	26847998	382999	327.268242	327.268124	-0.36
N	$C_{22}H_{33}O_2$	10656654	365106	329.247507	329.247367	-0.43
	$C_{24}H_{35}O$	29355636	365382	339.268242	339.268127	-0.34
AP	$C_{24}H_{37}O$	18161420	358362	341.283892	341.283732	-0.47
O	$C_{23}H_{35}O_2$	41643940	369087	343.263157	343.263035	-0.36
P	$C_{24}H_{37}O_2$	6802424	332705	357.278807	357.2786	-0.58
$[M+H]^+ - 3H_2O$	$C_{27}H_{39}$	1894413824	343871	363.304628	363.304715	0.24
$[M+H]^+$ -	$C_{27}H_{41}O$	3714219008	327414	381.315192	381.315269	0.20

Assignment	Elemental composition	Intensity	Resolution	Theoretical *m/z*	Observed *m/z*	Mass error (ppm)
$2H_2O$						
*$[M+H]^+$ - H_2O	$C_{27}H_{43}O_2$	4040327936	313373	399.325757	399.325759	0.01
$[M+H]^+$	$C_{27}H_{44}O_3$	66586640	303294	417.336322	417.336034	-0.69
**Average error**						0.04
**Absolute average error**						0.16
**Standard deviation**						0.15

Table S 2: Peak assignment table for the CID MS/MS of the protonated 24,25 dihydroxylated vitamin D_3 isomer. *Represents the peaks that were used for internal calibration.

Assignment	Elemental composition	Intensity	Resolution	Theoretical *m/z*	Observed *m/z*	Mass error (ppm)
AB	C_9H_{11}	1869928	1071496	119.085527	119.085556	0.24
	C_9H_{13}	12396664	1061040	121.101177	121.101208	0.26
	$C_8H_{13}O$	1181563	1089584	125.096091	125.096129	0.30
AC	$C_{10}H_{11}$	1677959	937313	131.085527	131.085554	0.21
	$C_{10}H_{13}$	2038017	889944	133.101177	133.101211	0.26
AD	$C_{11}H_{13}$	6366844	893308	145.101177	145.101194	0.12
	$C_{11}H_{15}$	6961119	883805	147.116827	147.116847	0.14
	$C_{11}H_{17}$	6275924	882670	149.132477	149.132509	0.21
AE	$C_{12}H_{15}$	12733265	829922	159.116827	159.11685	0.14
	$C_{13}H_{15}$	5637053	780588	171.116827	171.116848	0.12
AF	$C_{13}H_{17}$	14657403	741502	173.132477	173.132494	0.10
	$C_{13}H_{19}$	22800692	729735	175.148127	175.148134	0.04
	$C_{14}H_{15}$	2614706	648983	183.116827	183.116852	0.14
	$C_{14}H_{17}$	10953016	670327	185.132477	185.132503	0.14
*	$C_{14}H_{19}$	21418670	664199	187.148127	187.148127	0.00
	$C_{14}H_{21}$	21595156	662008	189.163777	189.163778	0.01
	$C_{15}H_{19}$	27630082	618707	199.148127	199.148133	0.03
	$C_{15}H_{21}$	34540292	609773	201.163777	201.163772	-0.02
AG	$C_{16}H_{21}$	37326840	573471	213.163777	213.163774	-0.01
	$C_{16}H_{23}$	34091172	561218	215.179427	215.179413	-0.07
	$C_{17}H_{21}$	16966516	541964	225.163777	225.163773	-0.02
AH	$C_{17}H_{23}$	37188592	540647	227.179427	227.179404	-0.10
*	$C_{17}H_{25}$	28857444	532460	229.195077	229.195077	0.00
	$C_{16}H_{25}O$	11507003	525096	233.189992	233.189972	-0.09
	$C_{18}H_{23}$	30439008	514971	239.179427	239.179425	-0.01
	$C_{18}H_{25}$	84186816	512230	241.195077	241.19504	-0.15
	$C_{18}H_{27}$	100554520	509859	243.210727	243.210708	-0.08
	$C_{18}H_{29}$	39622516	501056	245.226377	245.226365	-0.05
	$C_{17}H_{27}O$	10186701	488821	247.205642	247.205644	0.01
	$C_{19}H_{23}$	22505600	492517	251.179427	251.179396	-0.12
	$C_{19}H_{25}$	80828640	490115	253.195077	253.19503	-0.19
	$C_{19}H_{27}$	142137664	489376	255.210727	255.210663	-0.25
	$C_{18}H_{27}O$	70392304	478417	259.205642	259.205622	-0.08
	$C_{18}H_{29}O$	79927888	473736	261.221292	261.221258	-0.13
I	$C_{19}H_{27}O$	65494068	458462	271.205642	271.205596	-0.17
	$C_{19}H_{29}O$	174578832	457433	273.221292	273.221247	-0.16
	$C_{21}H_{27}$	52501960	446115	279.210727	279.210707	-0.07
	$C_{21}H_{29}$	70016560	443005	281.226377	281.226357	-0.07
	$C_{20}H_{27}O$	2186903	449643	283.205642	283.20561	-0.11
	$C_{21}H_{31}$	95444632	443805	283.242027	283.242002	-0.09

Assignment	Elemental composition	Intensity	Resolution	Theoretical *m/z*	Observed *m/z*	Mass error (ppm)
	$C_{20}H_{29}O$	73539328	439120	285.221292	285.221254	-0.13
	$C_{20}H_{31}O$	53714796	436218	287.236942	287.236927	-0.05
	$C_{20}H_{33}O$	5771226	430141	289.252592	289.252551	-0.14
	$C_{22}H_{31}$	339894560	424902	295.242027	295.241989	-0.13
J	$C_{21}H_{31}O$	73474568	418451	299.236942	299.236895	-0.16
	$C_{21}H_{33}O$	24789626	414105	301.252592	301.252594	0.01
	$C_{21}H_{35}O$	38273776	410898	303.268242	303.268233	-0.03
	$C_{23}H_{31}$	75506648	409239	307.242027	307.241997	-0.10
*	$C_{23}H_{33}$	60367956	403800	309.257677	309.257678	0.00
	$C_{23}H_{35}$	8760529	404676	311.273328	311.273337	0.03
K	$C_{22}H_{33}O$	30573900	398151	313.252592	313.252589	-0.01
	$C_{24}H_{33}$	71788360	389017	321.257677	321.257667	-0.03
	$C_{24}H_{35}$	147974096	388923	323.273328	323.273313	-0.05
	$C_{24}H_{37}$	1444946	425976	325.288978	325.288984	0.02
L	$C_{23}H_{35}O$	4925655	389572	327.268242	327.26822	-0.07
	$C_{24}H_{35}O$	23751706	369922	339.268242	339.268238	-0.01
	$C_{24}H_{37}O$	119270576	368247	341.283892	341.283874	-0.05
M	$C_{24}H_{37}O_2$	2125161	366485	357.278807	357.278835	0.08
$[M+H]^+ - 3H_2O$	$C_{27}H_{39}$	1050571584	346006	363.304628	363.304579	-0.13
$[M+H]^+ - 2H_2O$	$C_{27}H_{41}O$	3261233408	329163	381.315192	381.315092	-0.26
*$[M+H]^+ - H_2O$	$C_{27}H_{43}O_2$	640581440	313847	399.325757	399.325756	0.00
$[M+H]^+$	$C_{27}H_{44}O_3$	43943192	301238	417.336322	417.336392	0.17
**Average error**						0.01
**Absolute average error**						0.10
**Standard deviation**						0.08

Table S 3: Peak assignment table for the IRMPD MS/MS of the protonated 1,25 dihydroxylated vitamin D_3 isomer. *Represents the peaks that were used for internal calibration.

Assignment	Elemental composition	Intensity	Resolution	Theoretical *m/z*	Observed *m/z*	Mass error (ppm)
	C_8H_{15}	4141042	1223797	111.116827	111.116828	0.01
	C_9H_9	3162382	1207595	117.069877	117.069883	0.05
AB	C_9H_{11}	9180172	1110064	119.085527	119.085527	0.00
*	C_8H_9O	4794784	1067511	121.064791	121.064792	0.01
	C_9H_{13}	9434145	1027320	121.101177	121.101177	0.00
	$C_8H_{11}O$	1227280	1198025	123.080441	123.080442	0.01
	C_9H_{15}	13603153	1043694	123.116827	123.116833	0.05
	$C_7H_{10}O_2$	22377998	988679	127.075356	127.075359	0.02
	C_9H_9O	3407312	893098	133.064791	133.064785	-0.05
AD	$C_9H_{11}O$	61992800	912006	135.080441	135.080441	0.00
	$C_9H_{13}O$	14729875	886280	137.096091	137.09609	-0.01
*C	$C_8H_{11}O_2$	21955042	880931	139.075356	139.075355	-0.01
	$C_{11}H_{13}$	13500047	849301	145.101177	145.101166	-0.08
AE	$C_{10}H_{11}O$	2899916	841614	147.080441	147.080426	-0.10
	$C_{10}H_{13}O$	7497256	821052	149.096091	149.09609	-0.01
	$C_9H_{11}O_2$	35243024	814615	151.075356	151.075353	-0.02
D	$C_9H_{12}O_2$	6456592	830834	152.083181	152.083173	-0.05
	$C_9H_{13}O_2$	42139156	808019	153.091006	153.090996	-0.07
	$C_{12}H_{11}$	3405859	799996	155.085527	155.085512	-0.10
	$C_{12}H_{13}$	10882631	812535	157.101177	157.101162	-0.10
	$C_{12}H_{15}$	17157238	782252	159.116827	159.116821	-0.04
AF	$C_{11}H_{13}O$	3969191	760310	161.096091	161.096092	0.01
	$C_{12}H_{17}$	16109234	771371	161.132477	161.132468	-0.06
	$C_{11}H_{15}O$	5644011	764213	163.111742	163.111733	-0.06
	$C_{12}H_{19}$	44210420	765603	163.148127	163.148117	-0.06
E	$C_{10}H_{12}O_2$	7188778	770119	165.091006	165.091003	-0.02
	$C_{13}H_{15}$	22198802	734421	171.116827	171.116815	-0.07
	$C_{13}H_{17}$	21697632	716744	173.132477	173.132466	-0.06
AG	$C_{12}H_{15}O$	4840097	721043	175.111742	175.111733	-0.05
	$C_{13}H_{21}$	39102704	708411	177.163777	177.163773	-0.02
	$C_{14}H_{13}$	2501446	757234	181.101177	181.101175	-0.01
	$C_{14}H_{15}$	14540652	688350	183.116827	183.116816	-0.06
	$C_{14}H_{17}$	29294980	675596	185.132477	185.132474	-0.02
	$C_{14}H_{19}$	20597644	668850	187.148127	187.148114	-0.07
AH	$C_{13}H_{17}O$	11246969	677449	189.127392	189.12738	-0.06
	$C_{15}H_{15}$	5799643	655948	195.116827	195.116805	-0.11
	$C_{15}H_{17}$	19758214	633614	197.132477	197.132476	-0.01
	$C_{15}H_{19}$	35409440	626339	199.148127	199.148122	-0.03
	$C_{15}H_{21}$	21475238	622075	201.163777	201.163768	-0.04
	$C_{15}H_{23}$	30118174	614343	203.179427	203.179413	-0.07

Assignment	Elemental composition	Intensity	Resolution	Theoretical *m/z*	Observed *m/z*	Mass error (ppm)
CO	$C_{15}H_{25}$	8950406	609094	205.195077	205.195067	-0.05
	$C_{16}H_{17}$	10781976	602120	209.132477	209.132468	-0.04
	$C_{16}H_{19}$	22222418	594429	211.148127	211.148132	0.02
	$C_{16}H_{21}$	33290112	583929	213.163777	213.163777	0.00
	$C_{16}H_{23}$	25520288	578839	215.179427	215.179424	-0.01
	$C_{16}H_{25}$	34314676	574544	217.195077	217.195065	-0.06
CP	$C_{16}H_{27}$	15055544	564836	219.210727	219.210712	-0.07
	$C_{17}H_{19}$	10728598	560044	223.148127	223.148113	-0.06
	$C_{17}H_{21}$	31931770	549860	225.163777	225.163775	-0.01
	$C_{16}H_{19}O$	11527761	547202	227.143042	227.143041	0.00
AI	$C_{16}H_{21}O$	14839585	543657	229.158692	229.158679	-0.06
	$C_{17}H_{25}$	26799908	539293	229.195077	229.195084	0.03
	$C_{16}H_{23}O$	20008936	539591	231.174342	231.174326	-0.07
	$C_{17}H_{27}$	34766828	537008	231.210727	231.210734	0.03
	$C_{17}H_{29}$	13113514	527816	233.226377	233.226389	0.05
	$C_{18}H_{21}$	28041734	522105	237.163777	237.163785	0.03
	$C_{18}H_{23}$	35480236	518591	239.179427	239.179433	0.03
	$C_{18}H_{25}$	31261516	518563	241.195077	241.195069	-0.03
AJ	$C_{17}H_{23}O$	21914594	513123	243.174342	243.174322	-0.08
	$C_{18}H_{27}$	58065892	510607	243.210727	243.210737	0.04
	$C_{18}H_{29}$	156288128	507276	245.226377	245.226381	0.02
I	$C_{16}H_{22}O_2$	24454404	499315	247.169256	247.169252	-0.02
D'Q	$C_{18}H_{31}$	149341456	502566	247.242027	247.242018	-0.04
	$C_{19}H_{25}$	90932888	491510	253.195077	253.195064	-0.05
	$C_{19}H_{29}$	38602640	480314	257.226377	257.226373	-0.02
	$C_{19}H_{31}$	68454408	478619	259.242027	259.242016	-0.04
J	$C_{17}H_{24}O_2$	10025082	478826	261.184906	261.184886	-0.08
	$C_{18}H_{29}O$	10991740	476765	261.221292	261.221295	0.01
	$C_{18}H_{31}O$	24353010	468928	263.236942	263.236958	0.06
AL	$C_{19}H_{27}O$	82430648	457319	271.205642	271.20563	-0.04
B'Q	$C_{20}H_{33}$	89410344	452369	273.257677	273.25768	0.01
	$C_{18}H_{26}O_2$	8926107	443503	275.200557	275.20057	0.05
	$C_{21}H_{27}$	33991812	436608	279.210727	279.210731	0.01
	$C_{21}H_{29}$	39603452	434783	281.226377	281.226386	0.03
*	$C_{21}H_{31}$	23899508	427873	283.242027	283.242028	0.00
L	$C_{19}H_{27}O_2$	42282604	428683	287.200557	287.200563	0.02
	$C_{19}H_{29}O_2$	28569328	420913	289.216207	289.21622	0.04
	$C_{22}H_{31}$	6949008	429052	295.242027	295.242035	0.03
AM	$C_{21}H_{31}O$	20066750	404205	299.236942	299.236911	-0.10
	$C_{23}H_{31}$	73815136	397661	307.242027	307.242011	-0.05
	$C_{23}H_{33}$	28136736	393719	309.257677	309.257688	0.04
AN	$C_{22}H_{33}O$	1278894	331561	313.252592	313.252607	0.05
M	$C_{21}H_{31}O_2$	8755069	374332	315.231857	315.231813	-0.14
	$C_{21}H_{33}O_2$	9345112	390307	317.247507	317.247525	0.06

Assignment	Elemental composition	Intensity	Resolution	Theoretical *m/z*	Observed *m/z*	Mass error (ppm)
	$C_{24}H_{33}$	19081760	369762	321.257677	321.257666	-0.03
	$C_{24}H_{35}$	36993808	371881	323.273328	323.273328	0.00
	$C_{24}H_{37}$	17722374	368194	325.288978	325.288993	0.05
AO	$C_{23}H_{35}O$	15183102	363428	327.268242	327.268218	-0.07
N	$C_{22}H_{33}O_2$	3162620	388905	329.247507	329.247431	-0.23
	$C_{25}H_{37}$	21881404	353321	337.288978	337.288963	-0.04
	$C_{24}H_{35}O$	13845332	350171	339.268242	339.268217	-0.07
AP	$C_{24}H_{37}O$	13186168	342876	341.283892	341.283849	-0.13
O	$C_{23}H_{35}O_2$	7857562	348589	343.263157	343.263133	-0.07
P	$C_{24}H_{37}O_2$	2128920	356065	357.278807	357.278835	0.08
*$[M+H]^+$ - $3H_2O$	$C_{27}H_{39}$	827027968	341771	363.304628	363.30462	-0.02
$[M+H]^+$ - $2H_2O$	$C_{27}H_{41}O$	2130935296	326424	381.315192	381.315202	0.03
$[M+H]^+$ - H_2O	$C_{27}H_{43}O_2$	5138254336	314268	399.325757	399.325692	-0.16
$[M+H]^+$	$C_{27}H_{44}O_3$	263033312	294122	417.336322	417.336321	0.00
**Average error**						0.03
**Absolute average error**						0.05
**Standard deviation**						0.04

Table S 4: Peak assignment table for the IRMPD MS/MS of the protonated 24,25 dihydroxylated vitamin D_3 isomer. *Represents the peaks that were used for internal calibration.

Assignment	Elemental composition	Intensity	Resolution	Theoretical *m/z*	Observed *m/z*	Mass error (ppm)
	C_8H_{13}	32794890	1196925	109.101177	109.101222	0.41
AB	C_9H_{11}	12499045	1054654	119.085527	119.085564	0.31
	C_9H_{13}	144448384	1052295	121.101177	121.101209	0.26
	$C_8H_{13}O$	39061508	994911	125.096091	125.096125	0.27
	$C_8H_{15}O$	649833600	988496	127.111742	127.111758	0.13
AC	$C_{10}H_{11}$	14868465	922309	131.085527	131.085566	0.30
	$C_{10}H_{13}$	13019374	945840	133.101177	133.101205	0.21
	$C_{10}H_{15}$	61624340	917892	135.116827	135.116853	0.19
	$C_{11}H_{11}$	3722737	850705	143.085527	143.085542	0.10
AD	$C_{11}H_{13}$	34826168	853145	145.101177	145.101195	0.12
	$C_{11}H_{15}$	28377496	841503	147.116827	147.116845	0.12
	$C_{11}H_{17}$	46254992	821634	149.132477	149.132502	0.17
	$C_{12}H_{11}$	3924427	833354	155.085527	155.085545	0.12
	$C_{12}H_{13}$	23462916	796546	157.101177	157.101189	0.08
AE	$C_{12}H_{15}$	44738120	776744	159.116827	159.116842	0.09
	$C_{12}H_{17}$	64866452	775570	161.132477	161.132488	0.07
	$C_{12}H_{19}$	38963944	766225	163.148127	163.14814	0.08
	$C_{13}H_{13}$	9758154	744709	169.101177	169.101186	0.05
	$C_{13}H_{15}$	40191004	725762	171.116827	171.116834	0.04
AF	$C_{13}H_{17}$	52295268	721671	173.132477	173.132484	0.04
	$C_{13}H_{19}$	80894112	713751	175.148127	175.14813	0.02
	$C_{14}H_{17}$	55722736	671054	185.132477	185.132485	0.04
*	$C_{14}H_{19}$	69469384	666583	187.148127	187.148127	0.00
	$C_{14}H_{21}$	66283140	662398	189.163777	189.163776	-0.01
	$C_{15}H_{19}$	99904936	626481	199.148127	199.148128	0.01
	$C_{15}H_{21}$	72232656	621101	201.163777	201.163775	-0.01
AG	$C_{16}H_{21}$	111981648	585253	213.163777	213.163777	0.00
	$C_{16}H_{25}$	43210168	573491	217.195077	217.195068	-0.04
	$C_{17}H_{21}$	62961672	553554	225.163777	225.163775	-0.01
AH	$C_{17}H_{23}$	106985616	550762	227.179427	227.179418	-0.04
*	$C_{17}H_{25}$	77232400	542255	229.195077	229.195079	0.01
	$C_{16}H_{25}O$	24463878	537023	233.189992	233.189983	-0.04
	$C_{18}H_{23}$	111393656	520174	239.179427	239.179428	0.00
	$C_{18}H_{25}$	209632240	517834	241.195077	241.195065	-0.05
	$C_{18}H_{27}$	141704320	512010	243.210727	243.21073	0.01
	$C_{18}H_{29}$	135478528	507500	245.226377	245.226376	0.00
	$C_{19}H_{25}$	211566448	491770	253.195077	253.195061	-0.06
	$C_{19}H_{27}$	282148896	487686	255.210727	255.210708	-0.07
	$C_{19}H_{29}$	84312272	482227	257.226377	257.226366	-0.04
	$C_{18}H_{27}O$	81723792	481220	259.205642	259.205644	0.01

Assignment	Elemental composition	Intensity	Resolution	Theoretical *m/z*	Observed *m/z*	Mass error (ppm)
	$C_{18}H_{29}O$	54116944	475520	261.221292	261.22129	-0.01
	$C_{20}H_{27}$	176374960	465600	267.210727	267.21071	-0.06
	$C_{20}H_{29}$	169002384	462490	269.226377	269.226357	-0.07
I	$C_{19}H_{27}O$	88802920	458558	271.205642	271.205628	-0.05
	$C_{19}H_{29}O$	259344192	455485	273.221292	273.221287	-0.02
	$C_{21}H_{27}$	95635952	442722	279.210727	279.210725	-0.01
	$C_{21}H_{29}$	141118176	441552	281.226377	281.22638	0.01
	$C_{20}H_{29}O$	131976368	436108	285.221292	285.221282	-0.04
	$C_{20}H_{31}O$	61017504	430867	287.236942	287.236945	0.01
	$C_{20}H_{33}O$	6254222	436577	289.252592	289.252576	-0.06
*	$C_{22}H_{31}$	365646144	420677	295.242027	295.242024	-0.01
J	$C_{21}H_{31}O$	81548064	413280	299.236942	299.236919	-0.08
	$C_{21}H_{33}O$	18985990	405109	301.252592	301.252601	0.03
	$C_{21}H_{35}O$	18855146	403445	303.268242	303.268234	-0.03
	$C_{23}H_{31}$	116414112	402309	307.242027	307.242013	-0.05
	$C_{23}H_{33}$	56039292	397078	309.257677	309.257688	0.04
K	$C_{22}H_{33}O$	13269278	396106	313.252592	313.252599	0.02
	$C_{24}H_{33}$	67525688	379972	321.257677	321.257679	0.01
	$C_{24}H_{35}$	139746560	379937	323.273328	323.273331	0.01
L	$C_{23}H_{35}O$	1291381	435373	327.268242	327.268255	0.04
	$C_{24}H_{35}O$	14357176	350233	339.268242	339.268244	0.01
	$C_{24}H_{37}O$	51864428	353125	341.283892	341.28389	-0.01
$[M+H]^+$ - $3H_2O$	$C_{27}H_{39}$	996371264	339751	363.304628	363.304616	-0.03
$[M+H]^+$ - $2H_2O$	$C_{27}H_{41}O$	2313384448	325828	381.315192	381.315177	-0.04
*$[M+H]^+$ - H_2O	$C_{27}H_{43}O_2$	1206090240	308590	399.325757	399.325759	0.01
$[M+H]^+$	$C_{27}H_{44}O_3$	62603664	278062	417.336322	417.33637	0.12
**Average error**						0.04
**Absolute average error**						0.07
**Standard deviation**						0.09

Table S 5: Peak assignment table for the 193 nm UVPD MS/MS of the protonated 1,25 dihydroxylated vitamin D_3 isomer. *Represents the peaks that were used for internal calibration.

Assignment	Elemental composition	Intensity	Resolution	Theoretical *m/z*	Observed *m/z*	Mass error (ppm)
*	C_8H_9	13742154	1084022	105.069877	105.069877	0.00
AB	C_7H_9O	7533579	1062180	109.064791	109.064789	-0.02
	C_8H_{15}	5477098	1012224	111.116827	111.116824	-0.03
	C_9H_9	15969965	1046541	117.069877	117.069875	-0.02
	C_9H_{11}	55903916	1014701	119.085527	119.085522	-0.04
	C_8H_9O	5620035	1018142	121.064791	121.064787	-0.03
	C_9H_{13}	30283972	1002986	121.101177	121.101172	-0.04
	$C_8H_{11}O$	3166527	1049248	123.080441	123.080442	0.01
	C_9H_{15}	16803584	1010279	123.116827	123.116824	-0.02
B	$C_7H_{10}O_2$	3403886	1039053	127.075356	127.075349	-0.06
	C_9H_9O	11441856	950805	133.064791	133.064783	-0.06
AD	$C_9H_{11}O$	80283672	914365	135.080441	135.080437	-0.03
	$C_9H_{13}O$	29013398	905520	137.096091	137.096088	-0.02
C	$C_8H_{11}O_2$	4315962	968182	139.075356	139.075354	-0.01
	$C_{11}H_{13}$	40700184	865289	145.101177	145.101172	-0.03
AE	$C_{10}H_{11}O$	5923146	871874	147.080441	147.080431	-0.07
	$C_{10}H_{13}O$	8735650	848776	149.096091	149.096091	0.00
	$C_9H_{11}O_2$	23089162	830598	151.075356	151.075354	-0.01
*D	$C_9H_{12}O_2$	5398861	820900	152.083181	152.08318	-0.01
	$C_9H_{13}O_2$	14555797	817125	153.091006	153.091001	-0.03
	$C_{12}H_{11}$	13179184	821729	155.085527	155.085523	-0.03
	$C_{12}H_{13}$	28868582	801816	157.101177	157.101173	-0.03
	$C_{12}H_{15}$	40034988	791246	159.116827	159.116825	-0.01
AF	$C_{11}H_{13}O$	12022132	789447	161.096091	161.09609	-0.01
	$C_{12}H_{17}$	37172608	781012	161.132477	161.132475	-0.01
	$C_{11}H_{15}O$	5187670	791056	163.111742	163.111744	0.01
	$C_{12}H_{19}$	15282275	775113	163.148127	163.148126	-0.01
E	$C_{10}H_{12}O_2$	2351923	930356	165.091006	165.090986	-0.12
	$C_{13}H_{15}$	31032332	734899	171.116827	171.116827	0.00
	$C_{13}H_{17}$	24809220	731732	173.132477	173.132476	-0.01
AG	$C_{12}H_{15}O$	11332077	732250	175.111742	175.11174	-0.01
	$C_{14}H_{11}$	2253488	766817	179.085527	179.085508	-0.11
	$C_{14}H_{13}$	10928782	702038	181.101177	181.101176	-0.01
	$C_{14}H_{15}$	22586718	693794	183.116827	183.116827	0.00
	$C_{14}H_{17}$	26925088	684101	185.132477	185.132478	0.01
	$C_{14}H_{19}$	16456400	672931	187.148127	187.148128	0.01
AH	$C_{13}H_{17}O$	5110624	683570	189.127392	189.127396	0.02
	$C_{15}H_{13}$	1843524	756056	193.101177	193.101171	-0.03
	$C_{15}H_{15}$	16852128	649942	195.116827	195.116829	0.01
	$C_{15}H_{17}$	21890790	631269	197.132477	197.132479	0.01

Assignment	Elemental composition	Intensity	Resolution	Theoretical *m/z*	Observed *m/z*	Mass error (ppm)
	$C_{15}H_{19}$	22853914	633091	199.148127	199.148128	0.01
	$C_{15}H_{21}$	13421368	635914	201.163777	201.163781	0.02
	$C_{15}H_{23}$	6365509	635533	203.179427	203.179428	0.00
CO	$C_{15}H_{25}$	1789758	613906	205.195077	205.195107	0.15
	$C_{16}H_{17}$	15434469	596630	209.132477	209.132476	0.00
	$C_{16}H_{19}$	14826666	608144	211.148127	211.148133	0.03
	$C_{16}H_{21}$	12764766	596733	213.163777	213.163782	0.02
	$C_{16}H_{23}$	7185410	600084	215.179427	215.179431	0.02
	$C_{16}H_{25}$	7505302	585992	217.195077	217.195082	0.02
CP	$C_{16}H_{27}$	4112154	605066	219.210727	219.210723	-0.02
	$C_{16}H_{19}O$	4104325	586179	227.143042	227.143041	0.00
AI	$C_{16}H_{21}O$	3130308	574432	229.158692	229.158664	-0.12
	$C_{17}H_{25}$	5494218	544845	229.195077	229.195076	0.00
	$C_{16}H_{23}O$	5563127	530303	231.174342	231.174343	0.00
	$C_{17}H_{27}$	15667964	539172	231.210727	231.210733	0.03
	$C_{18}H_{23}$	9920324	529900	239.179427	239.179439	0.05
	$C_{18}H_{25}$	7086143	518414	241.195077	241.195084	0.03
AJ	$C_{17}H_{23}O$	6746412	520511	243.174342	243.174346	0.02
	$C_{18}H_{27}$	19552560	510063	243.210727	243.210741	0.06
	$C_{18}H_{29}$	66312728	509273	245.226377	245.226386	0.04
I	$C_{16}H_{22}O_2$	10736366	507724	247.169256	247.169264	0.03
D'Q	$C_{18}H_{31}$	68590328	502623	247.242027	247.242037	0.04
	$C_{19}H_{25}$	15773016	497725	253.195077	253.195084	0.03
	$C_{19}H_{29}$	11185875	491779	257.226377	257.226392	0.06
	$C_{19}H_{31}$	12033928	480198	259.242027	259.242035	0.03
J	$C_{17}H_{24}O_2$	7000114	493796	261.184906	261.184916	0.04
	$C_{18}H_{29}O$	8517685	485959	261.221292	261.221297	0.02
	$C_{18}H_{31}O$	52945120	472063	263.236942	263.236954	0.05
AL	$C_{19}H_{27}O$	32801638	456819	271.205642	271.205653	0.04
B'Q	$C_{20}H_{33}$	31007750	453624	273.257677	273.2577	0.08
	$C_{21}H_{29}$	9762418	432742	281.226377	281.226396	0.07
	$C_{21}H_{31}$	4469521	429681	283.242027	283.24204	0.05
*L	$C_{19}H_{27}O_2$	21755982	428818	287.200557	287.200571	0.05
	$C_{19}H_{29}O_2$	41707764	425396	289.216207	289.216223	0.06
	$C_{22}H_{31}$	1740028	432588	295.242027	295.24201	-0.06
AM	$C_{21}H_{31}O$	13532269	413723	299.236942	299.236951	0.03
	$C_{23}H_{31}$	16314276	406376	307.242027	307.242042	0.05
	$C_{23}H_{33}$	4263047	421120	309.257677	309.257707	0.10
*M	$C_{21}H_{31}O_2$	6241120	391991	315.231857	315.23185	-0.02
	$C_{21}H_{33}O_2$	9477224	388439	317.247507	317.247535	0.09
	$C_{24}H_{35}$	10581947	383473	323.273328	323.273353	0.08
	$C_{24}H_{37}$	5118188	391997	325.288978	325.288994	0.05
AO	$C_{23}H_{35}O$	7850529	388796	327.268242	327.268244	0.01

Assignment	Elemental composition	Intensity	Resolution	Theoretical *m/z*	Observed *m/z*	Mass error (ppm)
N	$C_{22}H_{33}O_2$	2713440	368619	329.247507	329.247471	-0.11
	$C_{24}H_{35}O$	2443857	387737	339.268242	339.268281	0.11
AP	$C_{24}H_{37}O$	8188888	347207	341.283892	341.283882	-0.03
O	$C_{23}H_{35}O_2$	6172003	371085	343.263157	343.263171	0.04
*$[M+H]^+$ - $3H_2O$	$C_{27}H_{39}$	453302144	333248	363.304628	363.30462	-0.02
$[M+H]^+$ - $2H_2O$	$C_{27}H_{41}O$	2120543232	318552	381.315192	381.315147	-0.12
$[M+H]^+$ - H_2O	$C_{27}H_{43}O_2$	1.1182E+10	307498	399.325757	399.325664	-0.23
$[M+H]^+$	$C_{27}H_{44}O_3$	2048201216	288484	417.336322	417.336247	-0.18
**Average error**						0.00
**Absolute average error**						0.04
**Standard deviation**						0.04

Table S 6: Peak assignment table for the 193 nm UVPD MS/MS of the protonated 24,25 dihydroxylated vitamin D_3 isomer. *Represents the peaks that were used for internal calibration.

Assignment	Elemental composition	Intensity	Resolution	Theoretical *m/z*	Observed *m/z*	Mass error (ppm)
	C_8H_9	17712800	1059487	105.069877	105.069944	0.64
	C_8H_{11}	23954304	963545	107.085527	107.085587	0.56
	C_8H_{13}	24980924	967380	109.101177	109.101235	0.53
	C_9H_9	5715644	893719	117.069877	117.069921	0.38
AB	C_9H_{11}	31213176	919667	119.085527	119.085572	0.38
	C_9H_{13}	68264080	925553	121.101177	121.101219	0.35
	$C_8H_{13}O$	7556916	902875	125.096091	125.096129	0.30
	$C_8H_{15}O$	19691976	871688	127.111742	127.111777	0.28
AC	$C_{10}H_{11}$	26288002	864176	131.085527	131.085561	0.26
	$C_{10}H_{13}$	38538416	866276	133.101177	133.101208	0.23
	$C_{10}H_{15}$	35796492	856483	135.116827	135.116855	0.21
	$C_{11}H_{11}$	14143775	829719	143.085527	143.08555	0.16
AD	$C_{11}H_{13}$	49407816	819100	145.101177	145.101197	0.14
	$C_{11}H_{15}$	45961620	803419	147.116827	147.116846	0.13
	$C_{11}H_{17}$	18154492	793348	149.132477	149.132496	0.13
	$C_{12}H_{11}$	5500855	821106	155.085527	155.085539	0.08
	$C_{12}H_{13}$	24680066	759477	157.101177	157.10119	0.08
AE	$C_{12}H_{15}$	63619420	750320	159.116827	159.116839	0.08
	$C_{12}H_{17}$	41079884	744070	161.132477	161.132488	0.07
	$C_{12}H_{19}$	9879364	731004	163.148127	163.14814	0.08
	$C_{13}H_{13}$	8218688	710857	169.101177	169.101184	0.04
	$C_{13}H_{15}$	23843668	706342	171.116827	171.116832	0.03
AF	$C_{13}H_{17}$	36017768	699663	173.132477	173.132482	0.03
	$C_{13}H_{19}$	25557372	695992	175.148127	175.148131	0.02
	$C_{14}H_{17}$	27445308	652846	185.132477	185.132478	0.01
*	$C_{14}H_{19}$	28957452	653374	187.148127	187.148126	-0.01
	$C_{14}H_{21}$	11576778	659773	189.163777	189.163774	-0.02
	$C_{15}H_{19}$	31545894	614969	199.148127	199.148125	-0.01
	$C_{15}H_{21}$	17646698	608502	201.163777	201.163772	-0.02
AG	$C_{16}H_{21}$	17848420	578889	213.163777	213.163767	-0.05
	$C_{16}H_{25}$	3725319	550113	217.195077	217.195077	0.00
	$C_{17}H_{21}$	8090119	546034	225.163777	225.163759	-0.08
AH	$C_{17}H_{23}$	10290032	538181	227.179427	227.179413	-0.06
*	$C_{17}H_{25}$	5512396	531586	229.195077	229.195081	0.02
	$C_{16}H_{25}O$	4806484	554746	233.189992	233.18998	-0.05
	$C_{18}H_{23}$	10001087	520395	239.179427	239.179417	-0.04
	$C_{18}H_{25}$	16190413	508650	241.195077	241.195068	-0.04
	$C_{18}H_{27}$	12840144	501549	243.210727	243.21072	-0.03
	$C_{18}H_{29}$	10198472	503112	245.226377	245.226375	-0.01
	$C_{19}H_{25}$	17187124	492462	253.195077	253.195063	-0.06

	$C_{19}H_{27}$	26493434	478186	255.210727	255.210712	-0.06
	$C_{19}H_{29}$	9011897	474156	257.226377	257.226371	-0.02
	$C_{18}H_{27}O$	15508332	475697	259.205642	259.205634	-0.03
	$C_{18}H_{29}O$	22930460	456872	261.221292	261.221288	-0.02
	$C_{20}H_{27}$	9348607	470450	267.210727	267.210708	-0.07
	$C_{20}H_{29}$	24659606	458857	269.226377	269.226365	-0.04
I	$C_{19}H_{27}O$	29632296	453861	271.205642	271.20563	-0.04
*	$C_{19}H_{29}O$	42338232	443223	273.221292	273.221287	-0.02
	$C_{21}H_{27}$	10482003	438130	279.210727	279.210727	0.00
	$C_{21}H_{29}$	8143322	450387	281.226377	281.226392	0.05
	$C_{20}H_{29}O$	23407330	425577	285.221292	285.22129	-0.01
	$C_{20}H_{31}O$	25021286	419411	287.236942	287.236942	0.00
	$C_{20}H_{33}O$	7351276	421495	289.252592	289.252588	-0.01
*	$C_{22}H_{31}$	42511748	413578	295.242027	295.242027	0.00
J	$C_{21}H_{31}O$	14384799	401733	299.236942	299.23693	-0.04
	$C_{21}H_{33}O$	5789493	419512	301.252592	301.252609	0.06
	$C_{21}H_{35}O$	10422222	405905	303.268242	303.268239	-0.01
	$C_{23}H_{31}$	7698702	391351	307.242027	307.24204	0.04
	$C_{23}H_{33}$	11489721	400825	309.257677	309.257698	0.07
K	$C_{22}H_{33}O$	3940607	388112	313.252592	313.252593	0.00
	$C_{24}H_{33}$	6899268	389133	321.257677	321.257699	0.07
	$C_{24}H_{35}$	34338592	372583	323.273328	323.273335	0.02
	$C_{24}H_{35}O$	7132963	366082	339.268242	339.268259	0.05
	$C_{24}H_{37}O$	32473164	350800	341.283892	341.283905	0.04
L	$C_{24}H_{37}O_2$	17230564	343238	357.278807	357.27882	0.04
$[M+H]^+$ - $3H_2O$	$C_{27}H_{39}$	271241088	333614	363.304628	363.30465	0.06
$[M+H]^+$ - $2H_2O$	$C_{27}H_{41}O$	1960107904	315056	381.315192	381.315208	0.04
*$[M+H]^+$ - H_2O	$C_{27}H_{43}O_2$	2450845952	300639	399.325757	399.325759	0.01
$[M+H]^+$	$C_{27}H_{44}O_3$	4.3386E+10	295929	417.336322	417.336044	-0.67
**Average error**						0.06
**Absolute average error**						0.11
**Standard deviation**						0.15

Table S 7: Peak assignment table for the 213 nm UVPD MS/MS of the protonated 1,25 dihydroxylated vitamin D_3 isomer. *Represents the peaks that were used for internal calibration.

Assignment	Elemental composition	Intensity	Resolution	Theoretical *m/z*	Observed *m/z*	Mass error (ppm)
*	C_8H_9	15672403	1151259	105.069877	105.069877	0.00
AB	C_7H_9O	5671770	1056906	109.064791	109.064791	0.00
	C_8H_{15}	6888588	1090361	111.116827	111.116824	-0.03
	C_9H_9	13430582	1011544	117.069877	117.069875	-0.02
	C_9H_{11}	49778484	990567	119.085527	119.085524	-0.03
*	C_8H_9O	5599569	999581	121.064791	121.064791	0.00
	C_9H_{13}	22951762	977536	121.101177	121.101176	-0.01
	$C_8H_{11}O$	2289688	938768	123.080441	123.080442	0.01
	C_9H_{15}	13836954	966363	123.116827	123.116825	-0.02
B	$C_7H_{10}O_2$	4159928	962405	127.075356	127.075358	0.02
	C_9H_9O	9463323	894363	133.064791	133.064794	0.02
AD	$C_9H_{11}O$	48670132	889956	135.080441	135.080442	0.01
	$C_9H_{13}O$	35000188	876406	137.096091	137.096094	0.02
C	$C_8H_{11}O_2$	2791150	823341	139.075356	139.075361	0.04
	$C_{11}H_{13}$	22749264	829030	145.101177	145.101181	0.03
AE	$C_{10}H_{11}O$	4133081	884212	147.080441	147.080446	0.03
	$C_{10}H_{13}O$	5150603	857740	149.096091	149.0961	0.06
	$C_9H_{11}O_2$	11786313	811135	151.075356	151.075359	0.02
*D	$C_9H_{12}O_2$	2610871	849275	152.083181	152.08318	-0.01
	$C_9H_{13}O_2$	18639658	788350	153.091006	153.091011	0.03
	$C_{12}H_{11}$	10544663	790102	155.085527	155.085534	0.05
	$C_{12}H_{13}$	16558361	770181	157.101177	157.101184	0.04
	$C_{12}H_{15}$	22596648	765381	159.116827	159.116834	0.04
AF	$C_{11}H_{13}O$	7331118	744543	161.096091	161.096102	0.07
	$C_{12}H_{17}$	25896764	752798	161.132477	161.132483	0.04
	$C_{11}H_{15}O$	2998087	744423	163.111742	163.111737	-0.03
	$C_{12}H_{19}$	13368918	745713	163.148127	163.148134	0.04
E	$C_{10}H_{12}O_2$	1414098	761802	165.091006	165.091028	0.13
	$C_{13}H_{15}$	16768625	714157	171.116827	171.116835	0.05
	$C_{13}H_{17}$	16210278	707726	173.132477	173.132486	0.05
AG	$C_{12}H_{15}O$	6639675	729354	175.111742	175.111749	0.04
	$C_{14}H_{11}$	3494311	693657	179.085527	179.085535	0.04
	$C_{14}H_{13}$	7539774	672597	181.101177	181.101187	0.06
	$C_{14}H_{15}$	12363443	672572	183.116827	183.116836	0.05
	$C_{14}H_{17}$	14874883	658403	185.132477	185.132482	0.03
	$C_{14}H_{19}$	12227371	654713	187.148127	187.148139	0.06
AH	$C_{13}H_{17}O$	3160862	625464	189.127392	189.12741	0.10
	$C_{15}H_{13}$	1939845	666314	193.101177	193.101185	0.04
	$C_{15}H_{15}$	9479680	636826	195.116827	195.116836	0.05
	$C_{15}H_{17}$	11967316	623179	197.132477	197.132486	0.05

Assignment	Elemental composition	Intensity	Resolution	Theoretical *m/z*	Observed *m/z*	Mass error (ppm)
	$C_{15}H_{19}$	12997762	613691	199.148127	199.148138	0.06
	$C_{15}H_{21}$	7969165	592310	201.163777	201.163788	0.05
	$C_{15}H_{23}$	4253304	602797	203.179427	203.179441	0.07
	$C_{16}H_{17}$	8211585	586414	209.132477	209.132492	0.07
	$C_{16}H_{19}$	9263358	574695	211.148127	211.14814	0.06
	$C_{16}H_{21}$	8128870	584929	213.163777	213.163793	0.08
	$C_{16}H_{23}$	3685306	617326	215.179427	215.179433	0.03
	$C_{16}H_{25}$	2767356	557563	217.195077	217.195104	0.12
CP	$C_{16}H_{27}$	1415982	569506	219.210727	219.210722	-0.02
	$C_{16}H_{19}O$	4412040	546302	227.143042	227.143056	0.06
AI	$C_{16}H_{21}O$	2011789	593723	229.158692	229.158665	-0.12
	$C_{17}H_{25}$	2292877	568617	229.195077	229.195095	0.08
	$C_{16}H_{23}O$	1421712	564422	231.174342	231.174373	0.13
	$C_{17}H_{27}$	2171024	583507	231.210727	231.210728	0.00
	$C_{18}H_{23}$	4945581	518261	239.179427	239.179442	0.06
	$C_{18}H_{25}$	4529859	485225	241.195077	241.195099	0.09
AJ	$C_{17}H_{23}O$	1498339	484873	243.174342	243.174346	0.02
	$C_{18}H_{27}$	5268196	490698	243.210727	243.210743	0.07
	$C_{18}H_{29}$	12161809	494207	245.226377	245.226387	0.04
I	$C_{16}H_{22}O_2$	2013515	507795	247.169256	247.16925	-0.02
D'Q	$C_{18}H_{31}$	6907725	488724	247.242027	247.242034	0.03
	$C_{19}H_{25}$	4780132	488605	253.195077	253.195092	0.06
	$C_{19}H_{29}$	2540403	442551	257.226377	257.226368	-0.03
	$C_{19}H_{31}$	3244561	495967	259.242027	259.242022	-0.02
J	$C_{17}H_{24}O_2$	1328823	483799	261.184906	261.1849	-0.02
	$C_{18}H_{31}O$	5919603	446807	263.236942	263.236967	0.09
AL	$C_{19}H_{27}O$	4820673	425219	271.205642	271.205643	0.00
B'Q	$C_{20}H_{33}$	4935604	457158	273.257677	273.257695	0.07
K	$C_{18}H_{26}O_2$	1314466	449077	275.200557	275.20055	-0.03
	$C_{21}H_{29}$	2633134	422861	281.226377	281.226386	0.03
*L	$C_{19}H_{27}O_2$	2932959	405613	287.200557	287.200568	0.04
	$C_{19}H_{29}O_2$	6208506	414032	289.216207	289.216208	0.00
AM	$C_{21}H_{31}O$	1698177	477998	299.236942	299.236952	0.03
	$C_{23}H_{31}$	2934216	414648	307.242027	307.242035	0.03
M	$C_{21}H_{31}O_2$	1232320	422446	315.231857	315.23174	-0.37
	$C_{21}H_{33}O_2$	1666228	454891	317.247507	317.247464	-0.14
	$C_{24}H_{35}$	1740154	392020	323.273328	323.273315	-0.04
AP	$C_{24}H_{37}O$	1661324	401187	341.283892	341.2839	0.02
*$[M+H]^+ - 3H_2O$	$C_{27}H_{39}$	57241780	310990	363.304628	363.304615	-0.04
$[M+H]^+ - 2H_2O$	$C_{27}H_{41}O$	2.58E+08	290981	381.315192	381.315144	-0.13
$[M+H]^+ - H_2O$	$C_{27}H_{43}O_2$	1.82E+09	274191	399.325757	399.325579	-0.45
$[M+H]^+$	$C_{27}H_{44}O_3$	3.58E+08	257406	417.336322	417.336218	-0.25
**Average error**						0.01

Assignment	Elemental composition	Intensity	Resolution	Theoretical *m/z*	Observed *m/z*	Mass error (ppm)
**Absolute average error**						0.06
**Standard deviation**						0.07

Table S 8: Peak assignment table for the 213 nm UVPD MS/MS of the protonated 24,25 dihydroxylated vitamin D_3 isomer. *Represents the peaks that were used for internal calibration.

Assignment	Elemental composition	Intensity	Resolution	Theoretical *m/z*	Observed *m/z*	Mass error (ppm)
	C_8H_9	32881288	993093	105.069877	105.069924	0.45
	C_8H_{11}	33115198	956632	107.085527	107.085572	0.42
	C_8H_{13}	43788144	942111	109.101177	109.101221	0.40
	C_9H_9	9479829	797628	117.069877	117.069922	0.38
AD	C_9H_{11}	36245648	851076	119.085527	119.085567	0.34
	C_9H_{13}	1.39E+08	886775	121.101177	121.101203	0.21
	$C_8H_{11}O$	1322552	998741	123.080441	123.080476	0.28
	$C_8H_{13}O$	13515112	780069	125.096091	125.096127	0.29
	$C_8H_{15}O$	38782480	794507	127.111742	127.111779	0.29
AE	$C_{10}H_{11}$	30194694	745352	131.085527	131.085563	0.27
	$C_{10}H_{13}$	43497820	678928	133.101177	133.101206	0.22
	$C_{10}H_{15}$	48274152	675628	135.116827	135.11686	0.24
	$C_{11}H_{11}$	14663380	678338	143.085527	143.085561	0.24
AF	$C_{11}H_{13}$	50556216	644653	145.101177	145.101206	0.20
	$C_{11}H_{15}$	54138816	661344	147.116827	147.116855	0.19
	$C_{11}H_{17}$	27239012	630114	149.132477	149.132508	0.21
	$C_{12}H_{11}$	7587517	625584	155.085527	155.085556	0.19
	$C_{12}H_{13}$	22403510	586650	157.101177	157.101202	0.16
AF	$C_{12}H_{15}$	74735800	571460	159.116827	159.116844	0.11
	$C_{12}H_{17}$	59220936	595874	161.132477	161.132497	0.12
	$C_{12}H_{19}$	13951708	581309	163.148127	163.148153	0.16
	$C_{13}H_{13}$	8712179	465072	169.101177	169.101198	0.12
	$C_{13}H_{15}$	21958396	443574	171.116827	171.116844	0.10
AH	$C_{13}H_{17}$	47961592	480775	173.132477	173.132481	0.02
	$C_{13}H_{19}$	35442920	455054	175.148127	175.148136	0.05
	$C_{14}H_{17}$	31116826	453005	185.132477	185.132478	0.01
*	$C_{14}H_{19}$	41693288	440129	187.148127	187.148127	0.00
	$C_{14}H_{21}$	19845776	442528	189.163777	189.163771	-0.03
	$C_{15}H_{15}$	4023556	471765	195.116827	195.116818	-0.05
	$C_{15}H_{17}$	14429588	418279	197.132477	197.132469	-0.04
	$C_{15}H_{19}$	37749508	427533	199.148127	199.148124	-0.02
*	$C_{15}H_{21}$	27561042	436697	201.163777	201.163777	0.00
	$C_{15}H_{23}$	6179202	402756	203.179427	203.179439	0.06
AI	$C_{16}H_{21}$	25207266	413414	213.163777	213.163764	-0.06
	$C_{17}H_{25}$	4449089	412414	229.195077	229.195061	-0.07
	$C_{18}H_{23}$	9479774	376462	239.179427	239.179408	-0.08
	$C_{19}H_{27}$	11292916	372238	255.210727	255.210742	0.06
	$C_{18}H_{27}O$	2620914	389771	259.205642	259.205649	0.03
	$C_{18}H_{29}O$	3801215	335258	261.221292	261.221264	-0.11
L	$C_{19}H_{27}O$	4223906	353120	271.205642	271.205602	-0.15

Assignment	Elemental composition	Intensity	Resolution	Theoretical *m/z*	Observed *m/z*	Mass error (ppm)
*	$C_{19}H_{29}O$	7267927	370413	273.221292	273.221293	0.00
	$C_{21}H_{29}$	3108691	332520	281.226377	281.226385	0.03
	$C_{20}H_{29}O$	3475175	334525	285.221292	285.221285	-0.02
	$C_{20}H_{33}O$	1850505	424272	289.252592	289.252642	0.17
	$C_{22}H_{31}$	12050177	331542	295.242027	295.242051	0.08
M	$C_{21}H_{31}O$	3499177	403852	299.236942	299.236933	-0.03
	$C_{21}H_{33}O$	1760126	423229	301.252592	301.252547	-0.15
*	$C_{23}H_{33}$	2759874	357867	309.257677	309.257675	-0.01
	$C_{24}H_{35}$	11633214	314116	323.273328	323.273397	0.21
$[M+H]^+ - 3H_2O$	$C_{27}H_{39}$	1.14E+08	289404	363.304628	363.304735	0.29
$[M+H]^+ - 2H_2O$	$C_{27}H_{41}O$	9.16E+08	277034	381.315192	381.315195	0.01
*$[M+H]^+ - H_2O$	$C_{27}H_{43}O_2$	1.3E+09	264409	399.325757	399.325758	0.00
$[M+H]^+$	$C_{27}H_{44}O_3$	1.43E+10	272661	417.336322	417.335761	-1.34
**Average error**						0.08
**Absolute average error**						0.16
**Standard deviation**						0.20

Table S 9: Peak assignment table for the EID MS/MS of the protonated 1,25 dihydroxylated vitamin D_3 isomer. *Represents the peaks that were used for internal calibration.

Assignment	Elemental composition	Intensity	Resolution	Theoretical *m/z*	Observed *m/z*	Mass error (ppm)
*	C_8H_9	21745554	1151374	105.069877	105.069877	0.00
AB	C_7H_9O	5964417	1105781	109.064791	109.064792	0.01
	C_9H_9	11830329	1049674	117.069877	117.06988	0.03
	C_9H_{11}	15875639	1043152	119.085527	119.085529	0.02
	C_8H_9O	3233805	1072666	121.064791	121.064791	0.00
B	$C_7H_{10}O_2$	4893909	1030521	127.075356	127.075358	0.02
	C_9H_9O	23503828	941431	133.064791	133.064793	0.02
AD	$C_9H_{11}O$	23789826	921376	135.080441	135.080445	0.03
	$C_9H_{13}O$	7391829	931763	137.096091	137.096097	0.04
C	$C_8H_{11}O_2$	6098119	899829	139.075356	139.075361	0.04
	$C_{11}H_{13}$	15155438	865594	145.101177	145.101182	0.03
AE	$C_{10}H_{11}O$	2339543	861627	147.080441	147.080436	-0.03
	$C_{10}H_{13}O$	3729634	866417	149.096091	149.0961	0.06
	$C_9H_{11}O_2$	34396920	831224	151.075356	151.075357	0.01
*D	$C_9H_{12}O_2$	102794256	822435	152.083181	152.083181	0.00
	$C_9H_{13}O_2$	18203950	821412	153.091006	153.091007	0.01
	$C_{12}H_{15}$	14861349	785008	159.116827	159.116829	0.01
AF	$C_{11}H_{13}O$	3069574	776242	161.096091	161.096087	-0.02
	$C_{11}H_{15}O$	2252282	763080	163.111742	163.11173	-0.07
E	$C_{10}H_{12}O_2$	2562152	793103	165.091006	165.091009	0.02
	$C_{13}H_{15}$	12708566	738795	171.116827	171.116827	0.00
	$C_{13}H_{17}$	11684174	727635	173.132477	173.13248	0.02
AG	$C_{12}H_{15}O$	1432756	795431	175.111742	175.111754	0.07
	$C_{13}H_{19}$	8393663	722879	175.148127	175.148132	0.03
	$C_{13}H_{21}$	9359911	697506	177.163777	177.163776	-0.01
	$C_{13}H_{23}$	5438596	702218	179.179427	179.179425	-0.01
	$C_{14}H_{17}$	11805476	682507	185.132477	185.132479	0.01
	$C_{14}H_{19}$	7968060	679133	187.148127	187.148134	0.04
	$C_{13}H_{17}O$	3351859	707028	189.127392	189.127388	-0.02
	$C_{14}H_{21}$	8966972	660049	189.163777	189.163777	0.00
	$C_{14}H_{23}$	7812389	669199	191.179427	191.179423	-0.02
	$C_{14}H_{25}$	1819381	692395	193.195077	193.195078	0.01
	$C_{15}H_{17}$	8963647	649317	197.132477	197.132482	0.03
	$C_{15}H_{19}$	10753998	624941	199.148127	199.14813	0.02
	$C_{15}H_{21}$	6139208	647897	201.163777	201.163775	-0.01
	$C_{15}H_{23}$	6394542	625112	203.179427	203.179428	0.00
CO	$C_{15}H_{25}$	2119169	606449	205.195077	205.195085	0.04
	$C_{16}H_{17}$	6609508	597781	209.132477	209.13248	0.01
	$C_{16}H_{19}$	6869892	615474	211.148127	211.148129	0.01
	$C_{16}H_{21}$	7071892	595982	213.163777	213.16378	0.01

Assignment	Elemental composition	Intensity	Resolution	Theoretical *m/z*	Observed *m/z*	Mass error (ppm)
	$C_{16}H_{23}$	4808084	589301	215.179427	215.179422	-0.02
	$C_{16}H_{25}$	6851207	591871	217.195077	217.195076	0.00
CP	$C_{16}H_{27}$	3515756	608398	219.210727	219.210726	0.00
	$C_{16}H_{19}O$	2532989	565614	227.143042	227.143049	0.03
	$C_{17}H_{23}$	6315648	549340	227.179427	227.179433	0.03
AI	$C_{16}H_{21}O$	2878762	536560	229.158692	229.158691	0.00
	$C_{17}H_{25}$	4610862	572738	229.195077	229.195075	-0.01
	$C_{16}H_{23}O$	3845072	575035	231.174342	231.174344	0.01
	$C_{18}H_{25}$	5171871	530008	241.195077	241.195059	-0.07
AJ	$C_{17}H_{23}O$	3675934	528060	243.174342	243.174345	0.01
	$C_{18}H_{27}$	11402016	520715	243.210727	243.210729	0.01
	$C_{18}H_{29}$	27061150	520987	245.226377	245.226381	0.02
I	$C_{16}H_{22}O_2$	4609046	519625	247.169256	247.169249	-0.03
D'Q	$C_{18}H_{31}$	32082970	504844	247.242027	247.242027	0.00
	$C_{19}H_{29}$	5105265	493262	257.226377	257.226377	0.00
	$C_{19}H_{31}$	6321896	483961	259.242027	259.24203	0.01
J	$C_{17}H_{24}O_2$	2180443	494168	261.184906	261.184897	-0.03
	$C_{18}H_{29}O$	1826141	541555	261.221292	261.22132	0.11
	$C_{18}H_{31}O$	8505301	484603	263.236942	263.236941	0.00
	$C_{19}H_{25}O$	10422584	472959	269.189992	269.189992	0.00
AL	$C_{19}H_{27}O$	12378546	464222	271.205642	271.205649	0.03
B'Q	$C_{20}H_{33}$	11559471	463605	273.257677	273.257673	-0.01
	$C_{21}H_{29}$	4465699	471764	281.226377	281.226371	-0.02
	$C_{21}H_{31}$	2718886	455237	283.242027	283.242009	-0.06
*L	$C_{19}H_{27}O_2$	6839726	445370	287.200557	287.200554	-0.01
	$C_{19}H_{29}O_2$	13039160	434836	289.216207	289.216206	0.00
	$C_{22}H_{29}$	4446035	440516	293.226377	293.226373	-0.01
AM	$C_{21}H_{31}O$	3420436	448597	299.236942	299.236934	-0.03
	$C_{23}H_{31}$	8723357	415981	307.242027	307.242026	0.00
	$C_{23}H_{33}$	2063947	428409	309.257677	309.257723	0.15
M	$C_{21}H_{31}O_2$	2054762	401182	315.231857	315.231882	0.08
	$C_{24}H_{35}$	3794304	423870	323.273328	323.273321	-0.02
	$C_{23}H_{33}O$	3541580	430028	325.252592	325.252612	0.06
	$C_{24}H_{37}$	2174544	408656	325.288978	325.289004	0.08
AO	$C_{23}H_{35}O$	2199328	411714	327.268242	327.268246	0.01
N	$C_{22}H_{33}O_2$	2251236	384755	329.247507	329.247513	0.02
	$C_{24}H_{35}O$	1669210	402380	339.268242	339.268286	0.13
AP	$C_{24}H_{37}O$	1953095	399336	341.283892	341.283854	-0.11
O	$C_{23}H_{35}O_2$	1248819	413526	343.263157	343.263223	0.19
*$[M+H]^{1+}$ - $3H_2O$	$C_{27}H_{39}$	102396960	346378	363.304628	363.304631	0.01
$[M+H]^{1+}$ - $2H_2O$	$C_{27}H_{41}O$	366968256	330378	381.315192	381.315188	-0.01
$[M+H]^{1+}$ - H_2O	$C_{27}H_{43}O_2$	1173587840	313586	399.325757	399.325738	-0.05
$[M+H]^{1+}$	$C_{27}H_{44}O_3$	350588896	299779	417.336322	417.336317	-0.01

Assignment	Elemental composition	Intensity	Resolution	Theoretical *m/z*	Observed *m/z*	Mass error (ppm)
**Average error**						0.01
**Absolute average error**						0.03
**Standard deviation**						0.03

Table S 10: Peak assignment table for the EID MS/MS of the protonated 24,25 dihydroxylated vitamin D_3 isomer. *Represents the peaks that were used for internal calibration.

Assignment	Elemental composition	Intensity	Resolution	Theoretical *m/z*	Observed *m/z*	Mass error (ppm)
	C_8H_9	18844638	1145651	105.069877	105.069888	0.10
	C_9H_9	7289019	1045181	117.069877	117.069884	0.06
AB	C_9H_{11}	22662456	1035656	119.085527	119.085536	0.08
	$C_8H_{13}O$	22515680	979822	125.096091	125.096103	0.10
	$C_8H_{15}O$	134854240	955111	127.111742	127.11175	0.06
AC	$C_{10}H_{11}$	21878770	940167	131.085527	131.085534	0.05
	$C_{10}H_{13}$	21992704	915657	133.101177	133.101181	0.03
	$C_{10}H_{15}$	31024698	900174	135.116827	135.116834	0.05
	$C_9H_{12}O$	1864736	984297	136.088266	136.088268	0.01
	$C_{11}H_{11}$	10245764	853042	143.085527	143.085531	0.03
	$C_8H_{15}O_2$	123945096	856517	143.106656	143.106661	0.03
AD	$C_{11}H_{13}$	33323628	843867	145.101177	145.101181	0.03
	$C_{11}H_{15}$	26104944	824577	147.116827	147.116831	0.03
	$C_{11}H_{17}$	21757580	825650	149.132477	149.132481	0.03
	$C_{12}H_{13}$	17960866	788574	157.101177	157.101179	0.01
AE	$C_{12}H_{15}$	37380624	769875	159.116827	159.116828	0.01
	$C_{12}H_{17}$	35484812	768739	161.132477	161.132477	0.00
	$C_{12}H_{19}$	13694730	761122	163.148127	163.148129	0.01
	$C_{13}H_{15}$	21231864	713370	171.116827	171.116827	0.00
AF	$C_{13}H_{17}$	30528370	707578	173.132477	173.132477	0.00
	$C_{13}H_{19}$	34203104	707453	175.148127	175.148127	0.00
	$C_{14}H_{15}$	9874170	687268	183.116827	183.116826	-0.01
	$C_{14}H_{17}$	26177814	660014	185.132477	185.132476	-0.01
*	$C_{14}H_{19}$	27771674	655968	187.148127	187.148127	0.00
	$C_{14}H_{21}$	22107396	655496	189.163777	189.163779	0.01
	$C_{15}H_{19}$	34209000	622183	199.148127	199.148122	-0.03
	$C_{15}H_{21}$	26725944	621027	201.163777	201.163774	-0.01
	$C_{15}H_{23}$	10372977	605300	203.179427	203.179424	-0.01
AG	$C_{16}H_{21}$	30389346	578700	213.163777	213.163772	-0.02
	$C_{16}H_{23}$	21472560	575191	215.179427	215.179422	-0.02
	$C_{16}H_{25}$	8388079	579923	217.195077	217.195076	0.00
	$C_{17}H_{21}$	13865070	551834	225.163777	225.163784	0.03
AH	$C_{17}H_{23}$	21498104	549497	227.179427	227.179424	-0.01
	$C_{17}H_{25}$	15128954	540581	229.195077	229.19507	-0.03
	$C_{16}H_{25}O$	5815914	527677	233.189992	233.189989	-0.01
	$C_{18}H_{23}$	19471284	518914	239.179427	239.179429	0.01
	$C_{18}H_{25}$	35908640	512497	241.195077	241.195072	-0.02

Assignment	Elemental composition	Intensity	Resolution	Theoretical *m/z*	Observed *m/z*	Mass error (ppm)
*	$C_{19}H_{27}$	48688972	484809	255.210727	255.210725	-0.01
	$C_{18}H_{27}O$	20942936	482884	259.205642	259.205635	-0.03
	$C_{18}H_{29}O$	21471460	481776	261.221292	261.221285	-0.03
I	$C_{19}H_{27}O$	23004112	464644	271.205642	271.205643	0.00
	$C_{19}H_{29}O$	41301104	457314	273.221292	273.221289	-0.01
	$C_{21}H_{29}$	19285780	441858	281.226377	281.226382	0.02
	$C_{20}H_{29}O$	24590454	438562	285.221292	285.221291	0.00
	$C_{20}H_{33}O$	3975654	444339	289.252592	289.252564	-0.10
*	$C_{22}H_{31}$	54287388	425696	295.242027	295.24203	0.01
J	$C_{21}H_{31}O$	14200228	421688	299.236942	299.236951	0.03
	$C_{21}H_{33}O$	4520557	430555	301.252592	301.252611	0.06
	$C_{23}H_{33}$	10482078	403587	309.257677	309.25769	0.04
K	$C_{22}H_{33}O$	2728770	417195	313.252592	313.252566	-0.08
	$C_{24}H_{35}$	29329280	387861	323.273328	323.273336	0.02
L	$C_{24}H_{37}O_2$	4873906	379840	357.278807	357.278775	-0.09
	$C_{24}H_{35}O$	1655922	353743	339.268242	339.268249	0.02
$[M+H]^{1+}$ - $3H_2O$	$C_{27}H_{39}$	215382672	342927	363.304628	363.304639	0.03
$[M+H]^{1+}$ - $2H_2O$	$C_{27}H_{41}O$	1077642368	323776	381.315192	381.315187	-0.01
*$[M+H]^{1+}$ - H_2O	$C_{27}H_{43}O_2$	1103859200	309031	399.325757	399.325755	-0.01
$[M+H]^{1+}$	$C_{27}H_{44}O_3$	8841828352	295556	417.336322	417.33614	-0.44
**Average error**						0.00
**Absolute average error**						0.04
**Standard deviation**						0.06

Table S 11: Fragmentation table for characteristic fragments, where one or both OH groups retained on the ring for 1,25$(OH)_2D_3$ which are absent in the 24,25$(OH)_2D_3$ MS/MS spectra.

1,25$(OH)_2D_3$ characteristic theoretical fragment (*m/z*)	Assignment	Fragmentation Method														
		CAD			IRMPD			EID			193 nm UVPD			213 nm UVPD		
		Intensity	S/N	Fragment to precursor intensity ratio (%)	Intensity	S/N	Fragment to precursor intensity ratio (%)	Intensity	S/N	Fragment to precursor intensity ratio (%)	Intensity	S/N	Fragment to precursor intensity ratio (%)	Intensity	S/N	Fragment to precursor intensity ratio (%)
109.064791	AB	X	X	X	X	X	X	medium	18.1	1.7	medium	53.6	0.37	medium	58	1.59
127.075356	B	X	X	X	high	232	8.51	medium	18.9	1.4	low	23.1	0.17	low	42	1.16
135.080441	AD	high	346.4	35.95	high	639	23.57	high	106.7	6.79	high	585.7	3.92	high	507	13.61
139.075356	C	low	26.8	2.98	high	224	8.35	medium	12.6	1.74	low	29.5	0.21	low	27.1	0.78
147.080441	AE	low	20	2.29	low	27.7	1.1	low	21.2	0.67	medium	40.9	0.29	low	40.6	1.16
152.083181	D	low	30.8	3.44	medium	63.6	2.45	high	42.2	29.32	medium	36.9	0.26	low	24.7	0.73
165.091006	E	low	44.2	4.93	medium	70.2	2.73	low	12.8	0.73	low	14.7	0.11	low	12.2	0.4
287.200557	L	high	390.1	499.33	high	423.9	16.07	medium	198.7	1.95	high	141.2	1.06	low	25.3	0.82
315.231857	M	high	27.2	40.38	medium	77.6	3.33	low	39.6	0.59	medium	38.3	0.3	low	9.2	0.34
329.247507	N	low	128.1	16	low	26.5	1.2	low	12.1	0.64	low	15.8	0.13	X	X	X
343.263157	O	high	493.1	27.27	medium	67.6	2.99	low	53.3	0.36	medium	36.9	0.3	X	X	X
357.278807	P	medium	78	10.22	low	16.7	0.81	X	X	X	X	X	X	X	X	X

a) **1,25-dihydroxyvitamin D₃ mass spectrum**

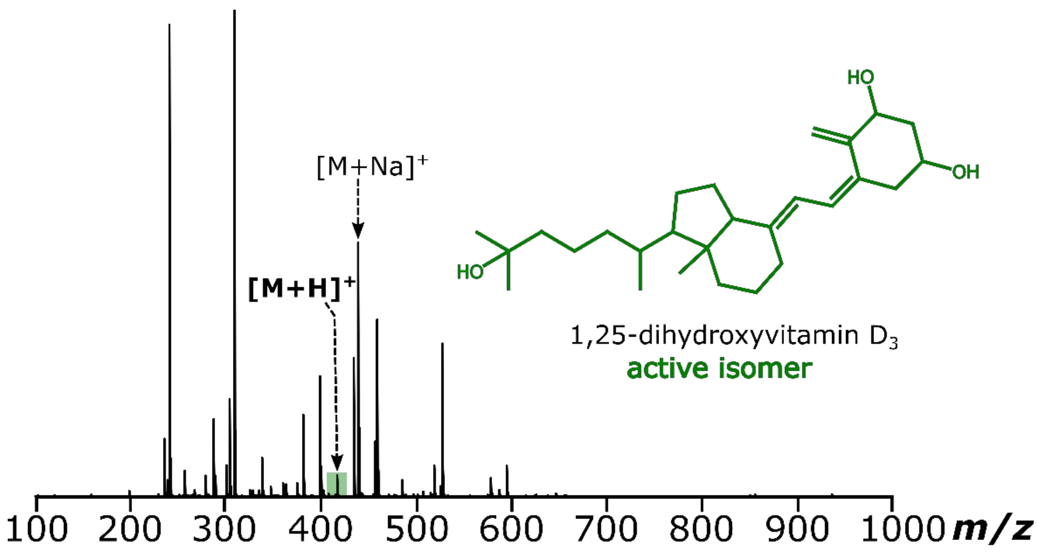

b) **24,25-dihydroxyvitamin D₃ mass spectrum**

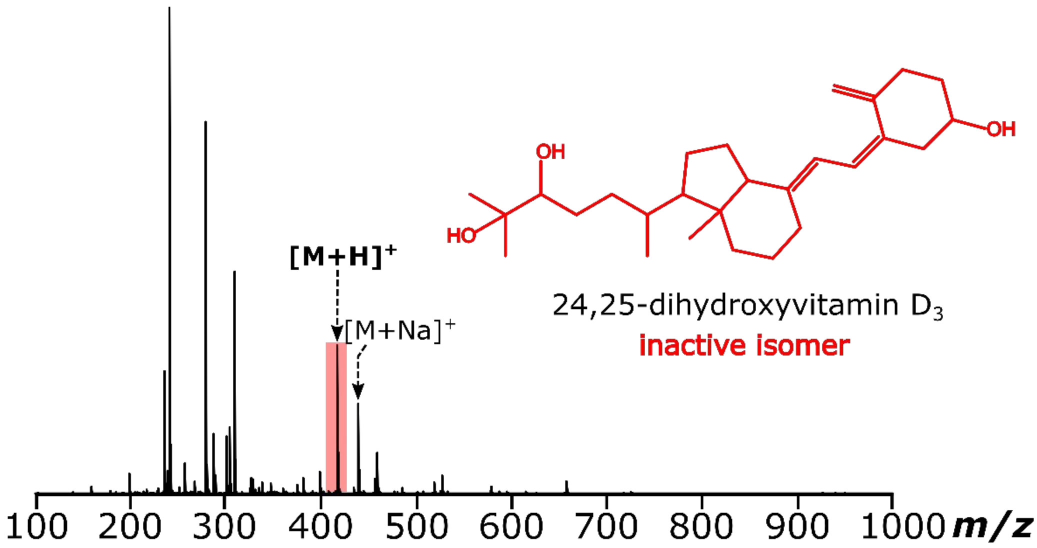

Figure S 1: Mass spectrum of a) 1,25-dihydroxylated vitamin D_3 and b) 24,25-dihydroxylated vitamin D_3.

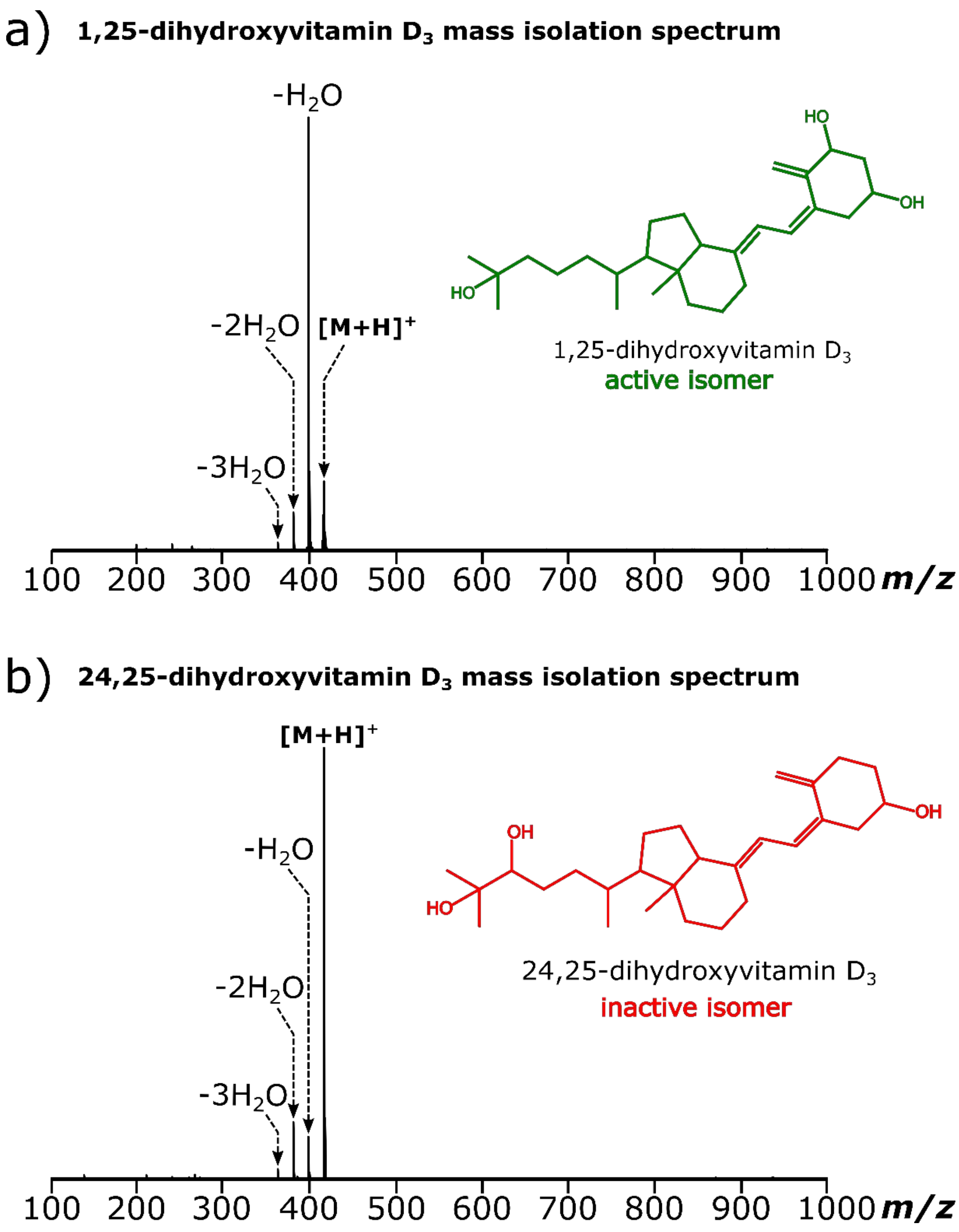

Figure S 2: Mass isolation spectrum with an *m/z* isolation window of 5 *m/z* for a) 1,25 dihydroxylated vitamin D_3 and b) 24,25 dihydroxylated vitamin D_3.

IRMPD MS/MS

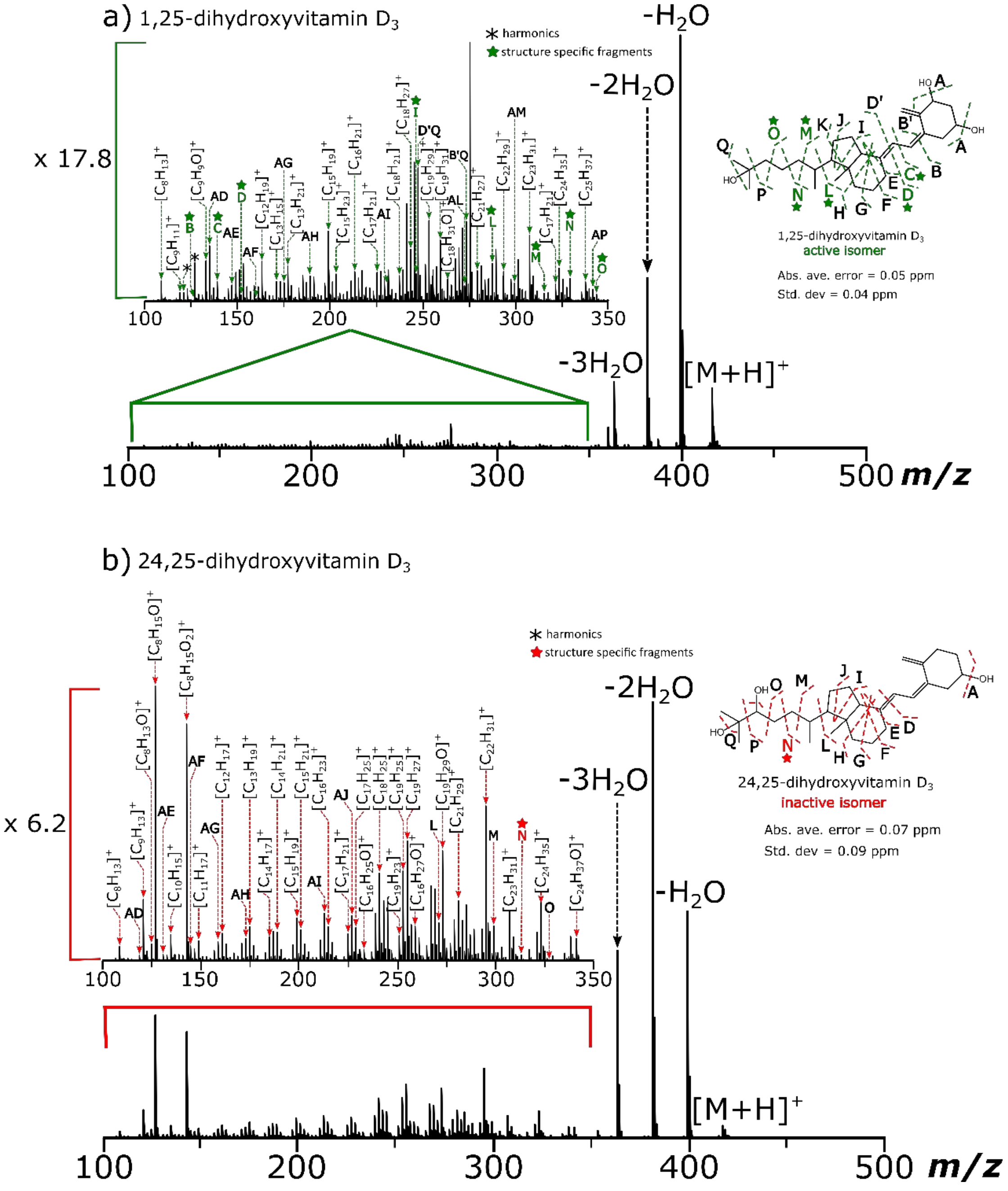

Figure S 3: IRMPD MS/MS spectra with inserts of *m/z* 100 – 350 regions with fragment peaks labelled for a) 1,25-dihydroxyvitamin D_3 and b) 24,25-dihydroxyvitamin D_3.

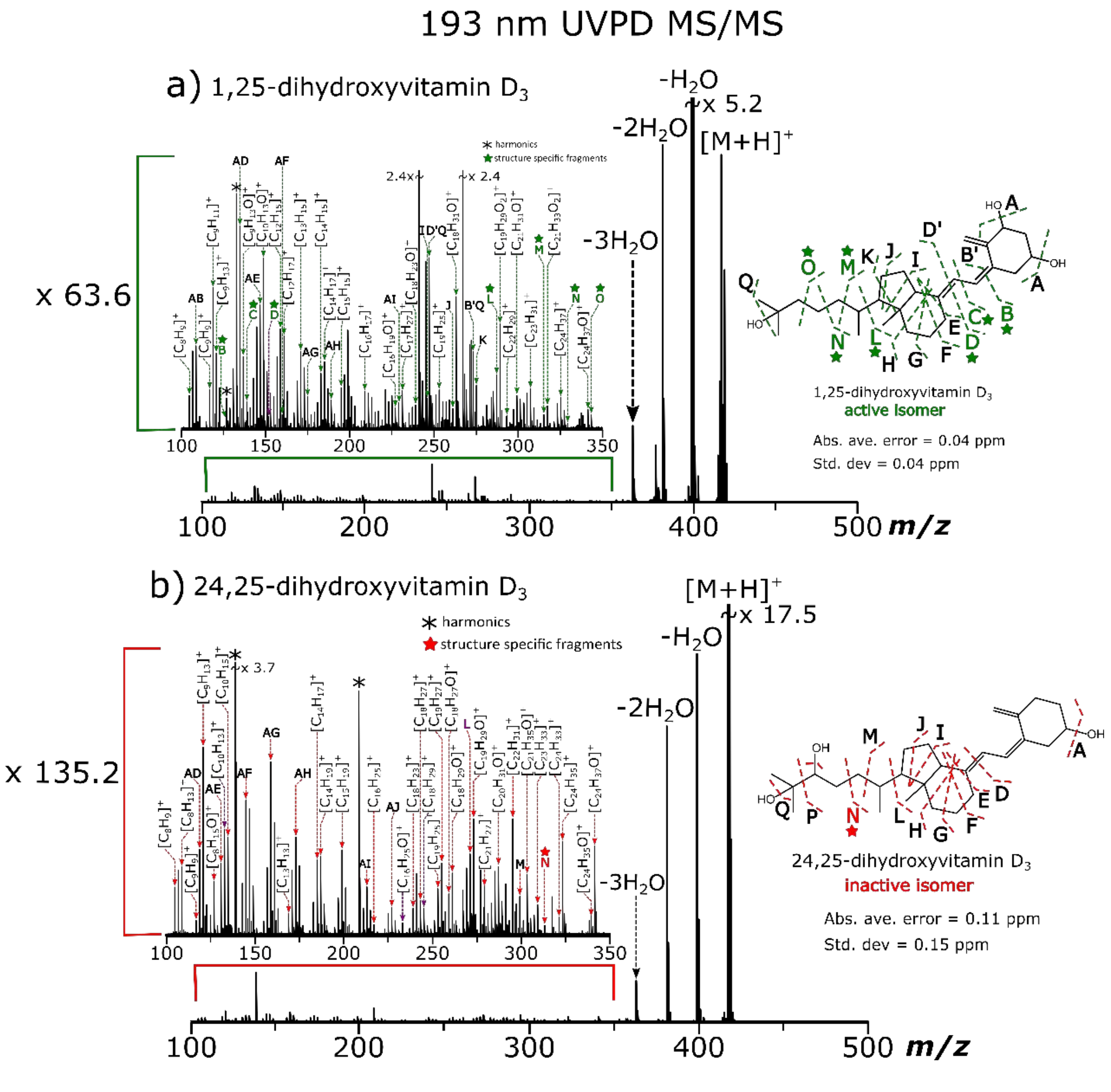

Figure S 4: 193 nm UVPD MS/MS spectra with inserts of *m/z* 100 – 350 regions with the fragment peaks labelled for a) 1,25-dihydroxyvitamin D_3 and b) 24,25-dihydroxyvitamin D_3.

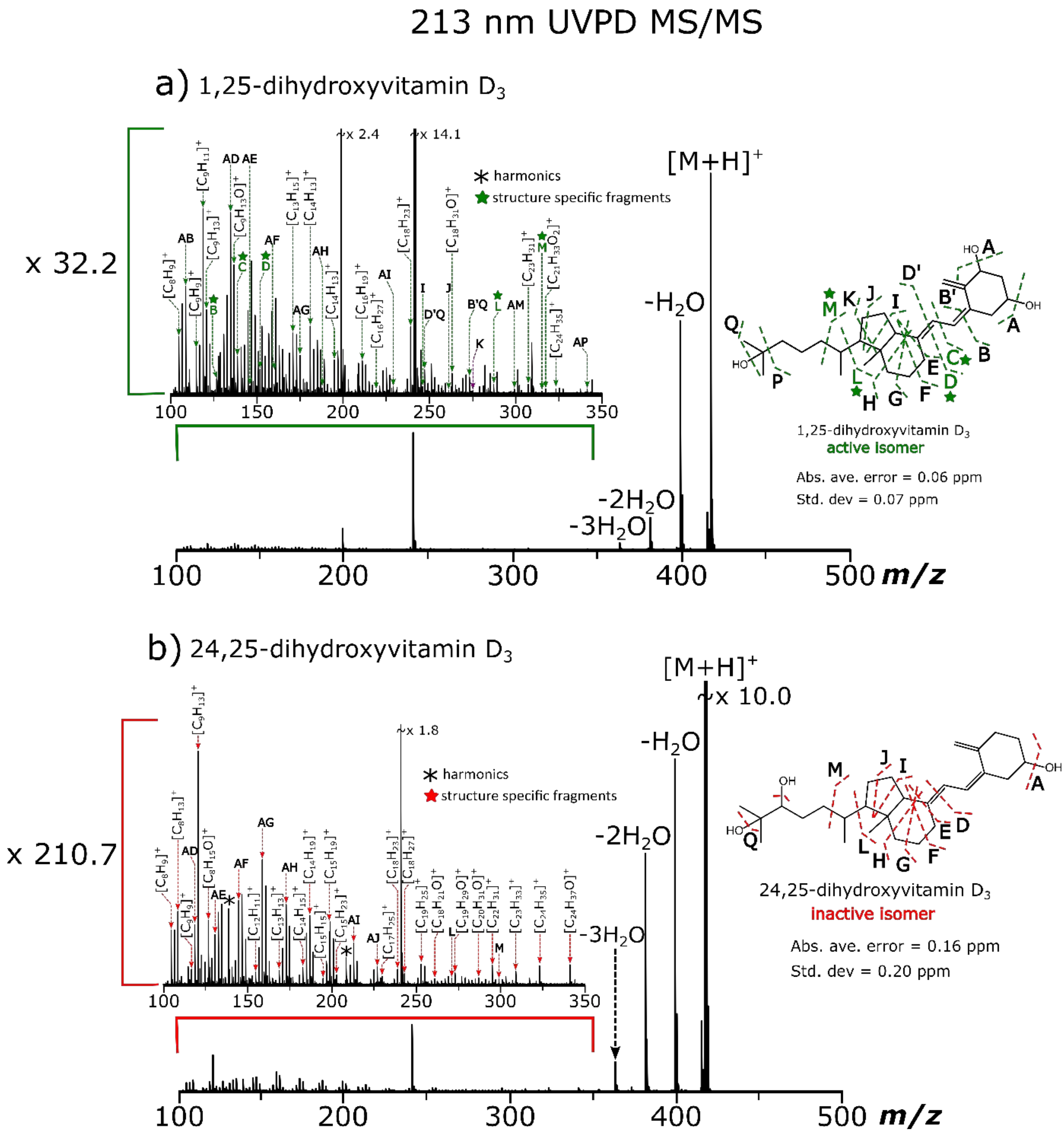

Figure S 5: 213 nm UVPD MS/MS spectra with inserts of *m/z* 100 – 350 regions with the fragment peaks labelled for a) 1,25-dihydroxyvitamin D_3 and b) 24,25-dihydroxyvitamin D_3.

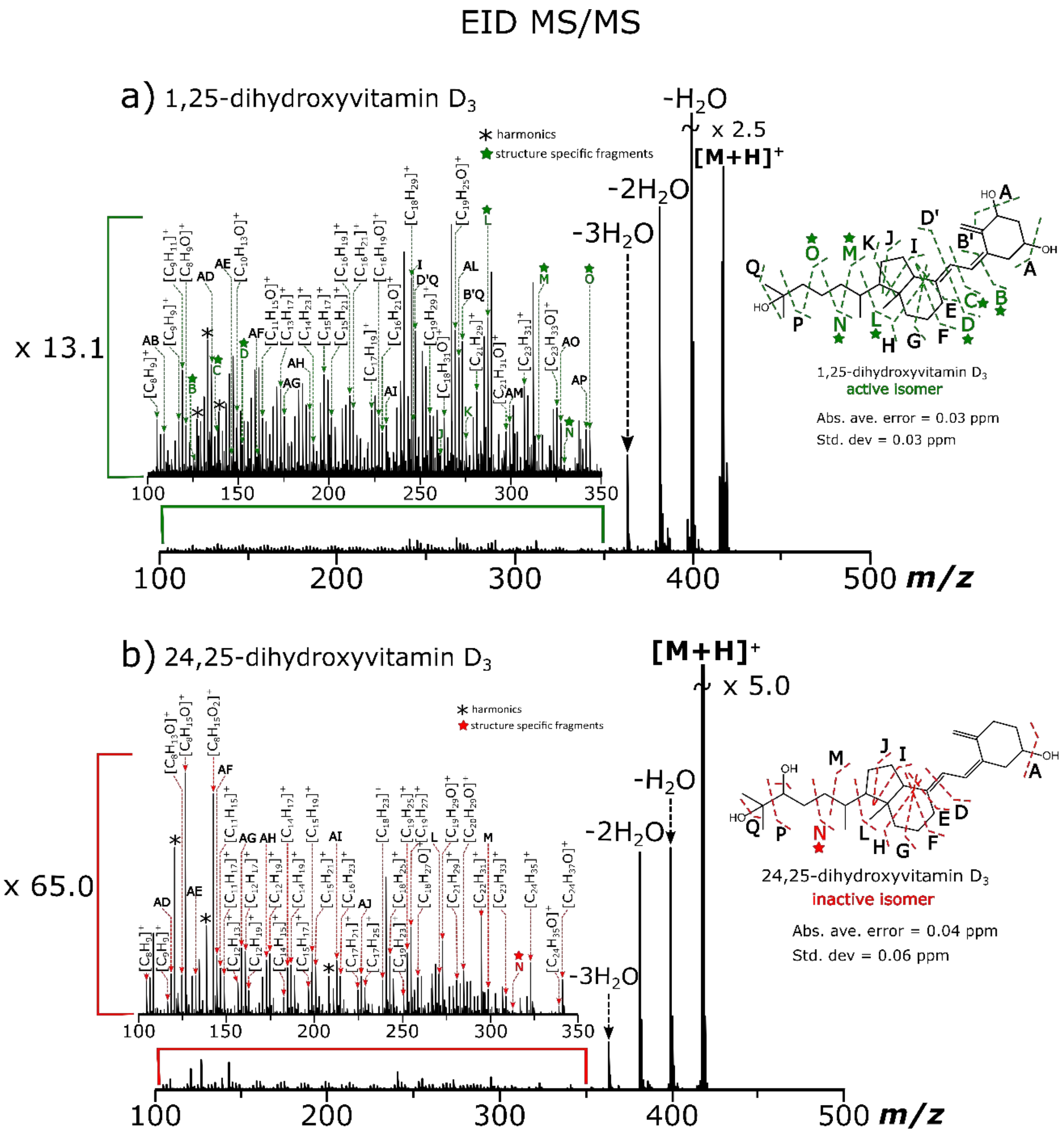

Figure S 6: EID MS/MS spectra with inserts of *m/z* 100 – 350 regions with fragment peaks labelled for a) 1,25-dihydroxyvitamin D_3 and b) 24,25-dihydroxyvitamin D_3.

Equation S 1: Equation to calculate the percentage fragmentation intensity to precursor intensity ratio for the characteristic fragments for 1,25-dihydroxyvitamin D_3.

$$Fragment\ to\ precursor\ intensity\ ratio\ (\%) = \frac{Fragment\ intensity}{Precursor\ intensity} \times 100$$
